# DeepTracer for fast de novo cryo-EM protein structure modeling and special studies on CoV-related complexes

**DOI:** 10.1073/pnas.2017525118

**Published:** 2020-12-23

**Authors:** Jonas Pfab, Nhut Minh Phan, Dong Si

**Affiliations:** ^a^Division of Computing and Software Systems, University of Washington Bothell, Bothell, WA 98011

**Keywords:** cryo-EM, de novo, modeling, complex, structure

## Abstract

Information about macromolecular structure of protein complexes and related cellular and molecular mechanisms can assist the search for vaccines and drug development processes. To obtain such structural information, we present DeepTracer, a fully automated deep learning-based method for fast de novo multichain protein complex structure determination from high-resolution cryoelectron microscopy (cryo-EM) maps. We applied DeepTracer on a previously published set of 476 raw experimental cryo-EM maps and compared the results with a current state of the art method. The residue coverage increased by over 30% using DeepTracer, and the rmsd value improved from 1.29 Å to 1.18 Å. Additionally, we applied DeepTracer on a set of 62 coronavirus-related cryo-EM maps, among them 10 with no deposited structure available in EMDataResource. We observed an average residue match of 84% with the deposited structures and an average rmsd of 0.93 Å. Additional tests with related methods further exemplify DeepTracer’s competitive accuracy and efficiency of structure modeling. DeepTracer allows for exceptionally fast computations, making it possible to trace around 60,000 residues in 350 chains within only 2 h. The web service is globally accessible at https://deeptracer.uw.edu.

## Significance

Electron cryomicroscopy (cryo-EM), a 2017 Nobel prize-awarded technology, provides direct 3D maps of macromolecules and explains the shape and interactions of protein complexes such as SARS-CoV-2 viral proteins and human cell receptors. This understanding can be combined with detailed structural information gathered using other technologies to form the basis for modeling course of diseases and for designing therapeutic drugs. However, ab initio modeling of protein complex structure remains a challenging problem. Here, we present DeepTracer, a fully automated and robust tool that determines the all-atom structure of a protein complex based solely on its cryo-EM map and amino acid sequence, with improved accuracy and efficiency compared to previous methods. We also provide a web service for global access.

The determining factor for a protein’s functionality is its structure, which is given by a unique sequence of amino acids and its three-dimensional (3D) arrangement (1). Consequently, researchers can draw conclusions about the behavior of the protein based solely on its molecular structure. This outcome can be useful in developing new vaccines and drugs, as viral fusion proteins play a central role in how the viruses invade the host’s cells (2). In order to prevent infections, researchers attempt to develop vaccines and medicines that target these fusion proteins. The structural information about the fusion proteins is crucial for researchers to predict their behaviors and ultimately to find the right vaccine (3).

To determine the structure of a protein, this work builds upon cryoelectron microscopy (cryo-EM) data (4). Cryo-EM allows researchers to capture macromolecules’ 3D maps at a near-atomic resolution. The technology has gained popularity in recent years as an alternative to established structure determination methods, such as X-ray crystallography, due to its improved quality and efficiency (5, 6). Amid the current global crisis, it is important that cryo-EM is being deployed right alongside X-ray crystallography to support the search for medicines and vaccines to fight the current COVID-19 pandemic (7). To derive the structure of a protein based on its 3D cryo-EM map, researchers currently either have to manually fit the atoms or resort to existing template-based or homology modeling methods (8–10). Therefore, there is a tremendous demand for a method that automatically and accurately determines the molecular structure from a cryo-EM map. Unfortunately, existing tools (11–15) such as Rosetta, MAINMAST (Mainchin model tracing from spanning tree), and Phenix determine only fragments of a protein complex, or require extensive manual processing steps. Due to the ability of cryo-EM to capture multiple large proteins in the course of a single study (16, 17), a fully automated, efficient tool to determine complex structures would be crucial to increase the throughput of the technology and speed up the development of medicines.

**Fig. 1.**
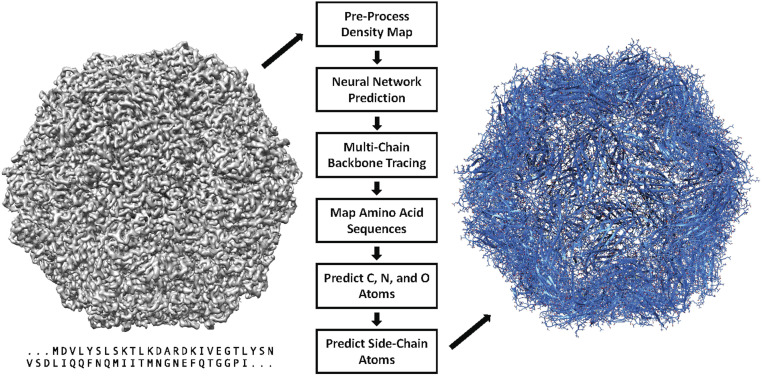
DeepTracer model determination pipeline. All-atom structure of multichain protein complexes is determined fully automatically solely from a cryo-EM map and amino acid sequence using the steps shown in the center of the figure. The structure shown on the right side is an actual model built by DeepTracer.

In this paper, we present DeepTracer, a fully automated software tool that determines the all-atom structure of a protein complex based solely on its cryo-EM map and amino acid sequence (Fig. 1). No manual processing of the map is necessary, and the tool requires no further parameters to run. The core of the method is a tailored deep convolutional neural network that allows for fast and accurate structure predictions when combined with complex preprocessing and postprocessing steps. This paper significantly improved our previous preliminary method and results (18). We also provide a web service and a CoV-related dataset along with the constructed models at DeepTracer’s website. This web service allows for fully automated protein complex determination and coronavirus modeling using 3D cryo-EM.

## Methods

DeepTracer performs an array of tasks to determine the structure of a protein. It preprocesses each cryo-EM map for the neural network, feeds the map to the network, and then transforms the output into a protein structure. An overview of the steps involved in this process is provided in Fig. 1. In this section, we focus on all steps, starting with the neural network. A detailed description of the preprocessing steps can be found in *SI Appendix*.

### Neural Network Architecture.

The convolutional neural network is the central piece of DeepTracer. Its job is to predict four vital pieces of information: the locations of amino acids, the location of the backbone, secondary structure positions, and amino acid types. Here, we take a closer look at the architecture of the neural network used in DeepTracer.

**Fig. 2.**
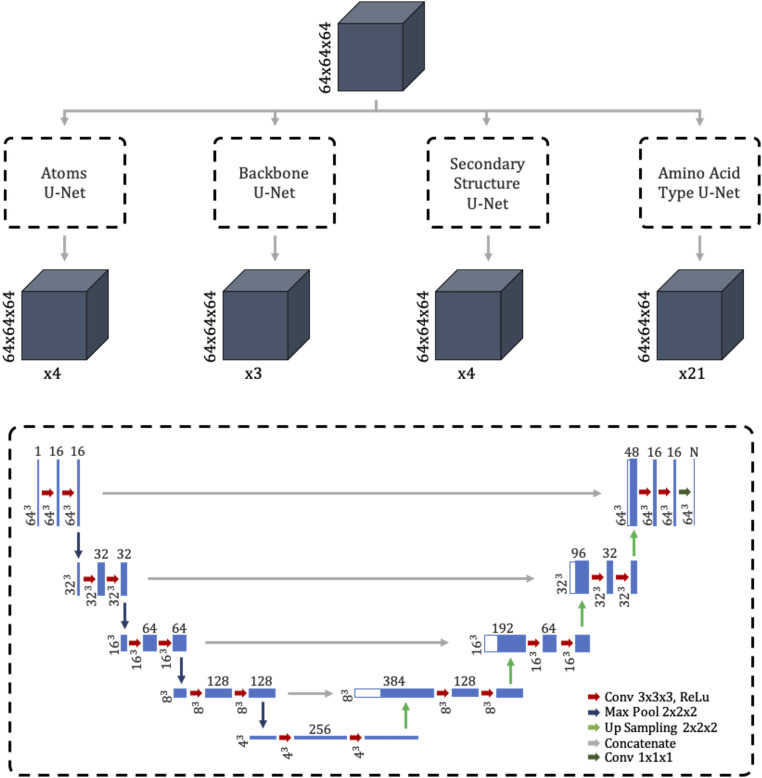
Architecture of tailored convolutional neural network. *Top* shows overview of DeepTracer’s neural network architecture consisting of four parallel U-Nets. The gray boxes show the input and output maps, with their dimensions noted to the left and the number of channels marked below. *Bottom* dashed box shows the detailed architecture of each parallel U-Net. The blue boxes show the output maps of the different layers, where the dimensions of the maps are depicted on the left and the number of channels is depicted on top.

The U-Net forms the basis for DeepTracer’s neural network. It is a convolutional network architecture developed by researchers at the University of Freiburg. Its name derives from the U shape of its architecture. The U-Net excels in fast and precise image segmentation tasks, particularly for biomedical applications (19). For DeepTracer, we modified its original 2D architecture for 3D cryo-EM maps and connected four separate U-Nets, one for each structural aspect (atoms, backbone, secondary structure elements, and amino acid types). The detailed architecture of the network used by DeepTracer can be seen in Fig. 2. The preprocessed cryo-EM maps are fed to the 64^3^ input layer of each U-Net. The output layer of each U-Net has the same 64^3^ shape, with a varying number of channels depending on which structural aspect it predicts. The following paragraph describes the output channels in detail.

The overall convolutional network consists of four U-Nets. The first U-Net is the Atoms U-Net, which determines whether each voxel contains either a Cα atom, a nitrogen atom, a carbon atom, or no atom. Thus, its output has four channels. The second U-Net is the Backbone U-Net, which determines whether each voxel belongs to the backbone, meaning either it is on the backbone, a part of a side chain, or not a part of the protein. Thus, it has three different output channels. Next, the Secondary Structure U-Net is responsible for finding out the secondary structure of each voxel. It has four output channels for loops, sheets, helices, and no structure. Finally, the Amino Acid Type U-Net determines the amino acid type for each voxel. As 20 different types of amino acids have been found in nature, this U-Net has 21 output channels, representing the amino acids plus the case in which the voxel is not part of the protein. By combining the separate U-Nets into a single network with four outputs, we can train it in a single run while specifying different loss functions for each output. Particularly, we used a weighted cross-entropy loss function for each U-Net, with a different set of weights depending on the class balance of its training data.

### Training Data Collection.

Before training the U-Net model, we have to collect a training dataset. Previous projects, such as ref. 14, used simulated cryo-EM maps to train their neural networks. However, for the network to learn common noise patterns in cryo-EM maps, we decided to use experimental maps. The maps were downloaded from the EMDataResource website (20), together with their deposited model structures that served as the ground truth in the training process and were fetched from Protein Data Bank (21). As this work focuses on high-resolution maps, we only used maps with a resolution of 4 Å or better. In total, we downloaded 1,800 experimental maps and their corresponding deposited model structures. The maps were randomly split into training and validation sets, with an 80:20 ratio.

To label each cryo-EM map, we created masks with the same dimensions as the grid of the map, providing a label for each voxel. The labels of the masks were hereby created based on the deposited model structures of each map. As shown in Fig. 2, the model has four different outputs, for each of which we created separate masks. The atoms mask provides a label for each voxel as to whether or not it contains a Cα, C, or N atom. Therefore, we filtered out these atoms from the protein structure, calculated the corresponding grid indices for their location, and set that voxel and all directly neighboring voxels to the value representing the atom (one for Cα, two for C, and three for N atoms). A visualization of an atom mask can be found in *SI Appendix*, Fig. S5.

**Fig. 3.**
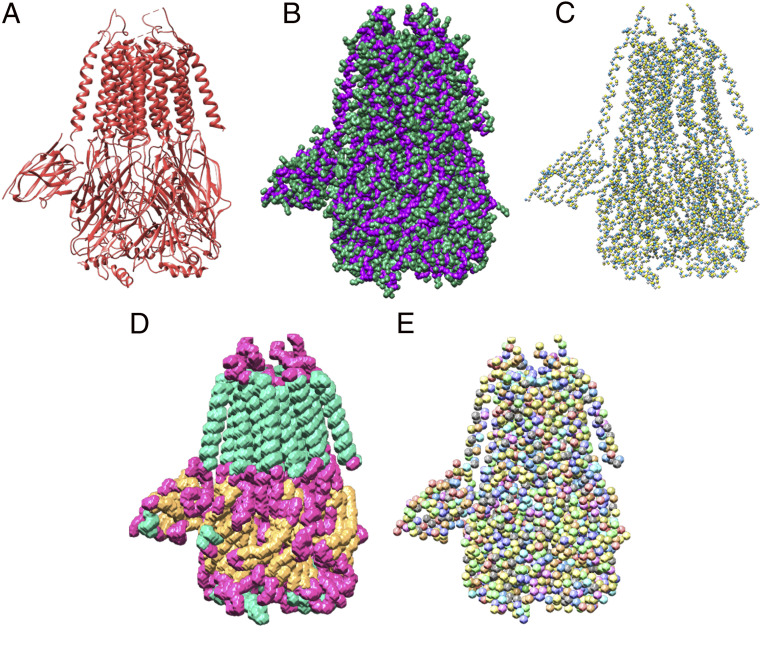
Example masks from the training dataset based on the PDB ID code 6NQ1 deposited model structure. (*A*) Deposited model structure. (*B*) Backbone (Cα, C, and N atoms) in purple and side chains in green. (*C*) Atoms mask with labels for Cα, C, and N atoms. (*D*) Secondary structure mask with helices in turquoise, loops in pink, and sheets in orange. (*E*) Amino acid type mask with 20 different colors.

The masks for the backbone, secondary structure, and Amino Acid Type U-Net were created in a similar manner. The backbone mask filters all backbone atoms and side-chain atoms and sets the respective voxels and all surrounding voxels with a distance of two to one for backbone and two for side chain. To create the secondary structure mask, we filtered all atoms for helices, sheets, and loops and then set all voxels with a distance of four surrounding the atoms to one for loop, two for helix, and three for sheet. Finally, for the amino acid type mask, all Cα atoms for each of the 20 amino acid types were filtered out, and all surrounding voxels within a distance of three were set to a value between 1 and 20, where each value corresponds to a specific amino acid type. An example of all masks can be seen in Fig. 3. An example of a raw prediction from the trained neural network for the EMD-6272 map can be found in *SI Appendix*, Fig. S4.

### Tracing Backbone.

This step uses the output of the U-Net to create an initial model structure that contains only Cα atoms connected into chains. This is a central postprocessing step, and its accuracy determines, to a great extent, how well the remaining postprocessing steps will perform. The step can be split into three different parts: identifying disconnected chains, which can be processed independently; calculating the *x*, *y*, and *z* coordinates of the Cα atoms; and connecting the Cα atoms into chains by applying a modified traveling salesman algorithm.

**Fig. 4.**
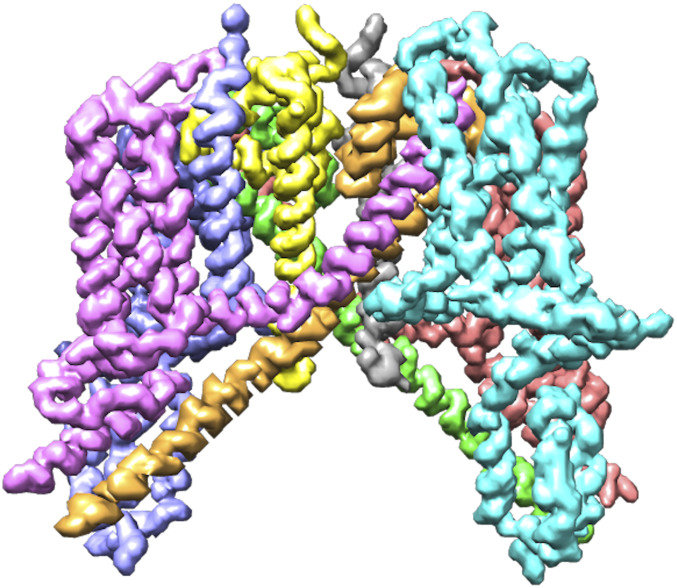
Backbone confidence map of the EMD-0478 map with identified chains annotated in different colors.

Identifying chains prior to any atom prediction has two advantages. First, it improves the performance of the step, as each chain will contain a lower number of atoms that have to be connected by the traveling salesman algorithm. Second, it decreases the number of incorrect connections between atoms of separate chains, as they are processed independently. To identify chains, we used the output of the Backbone U-Net. We rounded each voxel of the confidence map to either zero or one and then found connected areas of voxels with a value of one. Disconnected areas were then identified as separate chains. An example of the chain identification process visualized for the EMD-0478 map can be seen in Fig. 4.

To find the *x*, *y*, and *z* coordinates of the Cα atoms, we utilized the Cα channel from the output of the Atoms U-Net. A voxel value in this map describes the confidence of whether this voxel contains a Cα atom. The coordinates were then calculated in two steps. First, we found the indices of all local maximums in the confidence map within a distance of four voxels that have a minimum value of 0.5. Next, we refined the indices by calculating the center of mass of all voxels within a distance of four surrounding the local maximums. This is possible as we moved away from integer indices toward floating point coordinates, giving us the opportunity to express locations more precisely.

The most challenging part of this step is to connect the placed Cα atoms into chains correctly. The factorial growth of the number of ways in which the atoms can be connected makes it infeasible to test all possible solutions even for a low number of atoms. Therefore, we decided to solve the problem using an optimization algorithm, particularly, for the traveling salesman problem (TSP). However, our problem does not match every criterion of the TSP. The shortest possible path is not necessarily the correct one, as the ideal distance between Cα atoms is 3.8 Å (22). Deviations from this value are, however, possible due to prediction inaccuracies. Additionally, it is often difficult to decide, only based on the distance, which atoms to connect if there are multiple possibilities with a similar distance. To address these issues, we developed a custom confidence function instead of solely relying on the Euclidean distance between atoms. The confidence function’s idea is to return a score between zero and one, which expresses how confident we are that these two atoms are connected. The goal of the TSP algorithm is then to connect the atoms such that the sum of all confidence scores between connected atoms is maximized.

The calculation of the confidence score between Cα atoms considers two factors: the Euclidean distance between the atoms and the average density values of voxels that lay in between the atoms on the backbone confidence map predicted by the Backbone U-Net. The latter factor is to ensure that connections are made along the backbone of the structure. The voxels that lay between the atoms are found using Bresenham’s algorithm (23). To transform these metric values to a confidence score, we used a probability density function p(x,μ,σ) with a mean μ, which represents the ideal metric value, and a standard deviation σ. To make sure that the function returns exactly one at the mean, we normalized it by dividing it by the probability density value at the mean. For the Euclidean distance, we used a mean of 3.8 and a standard deviation of one. The average backbone confidence has a mean of one and a standard deviation of 0.3. The standard deviations were determined based on several rounds of testing. Both probability density functions can be seen in *SI Appendix*, Fig. S6. In order to combine both results into a single confidence score, we simply multiply both values. As the TSP algorithm was designed to minimize distances between paths, we then just subtract the confidence score from one and provide it to the algorithm.

**Fig. 5.**
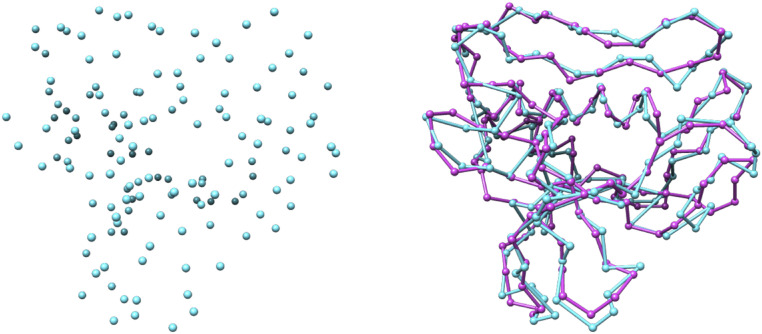
Traced backbone atoms. Predicted Cα atoms for the EMD-4054 map in blue before (*Left*) and after (*Right*) the backbone tracing step compared to the deposited model structure in pink.

To apply the TSP algorithm, we had to specify a start/end point. However, we could not know yet at which atom the chain will start and end. Therefore, we added a new atom that is connected to every other atom with a confidence of one. This atom was then specified as the start/end and, later on, removed from the actual chain. An example of the application of the TSP on a list of Cα atoms can be seen in Fig. 5.

### Amino Acid Sequence Mapping.

**Fig. 6.**
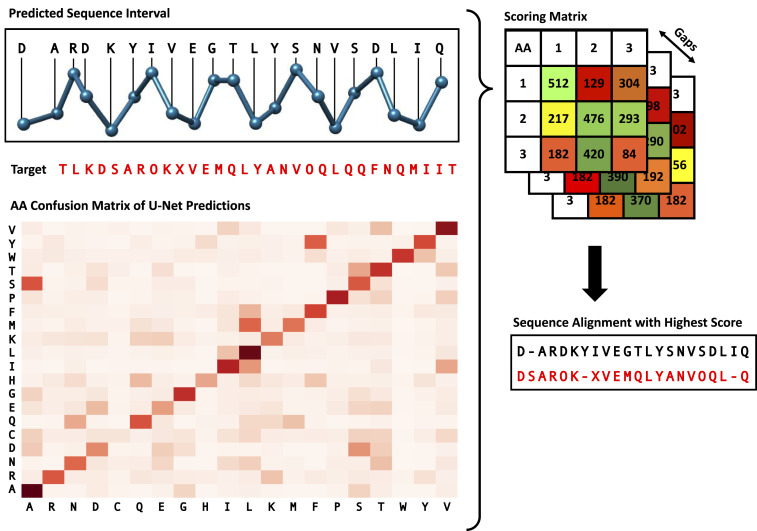
Protein sequence alignment algorithm. Interval of the predicted sequence is aligned with the target sequence using a custom dynamic algorithm. The amino acid confusion matrix depicts the relative frequency of pairs of predicted and true amino acid type and was calculated based on a set of test cryo-EM maps. The numbers shown in the score matrix are solely for illustrative purposes and do not reflect real data.

To realize the side-chain prediction for the protein structure, we first need to know each amino acid’s type. As discussed in *Neural Network Architecture*, one output of the deep learning model is the amino acid type prediction. However, depending on the resolution of the cryo-EM map, this prediction is of limited accuracy, around 10 to 50%, since some amino acids have a similar appearance in cryo-EM maps. The goal of this step is to improve the amino acid type accuracy by aligning intervals of the initially predicted sequence to the known true amino acid sequence (protein primary structure) and then updating the types of the predicted amino acids accordingly (Fig. 6).

Aligning amino acid sequences is a common problem in the field of bioinformatics, and previous research has led to the development of multiple algorithms (24–26). However, these algorithms are usually applied between different proteins to measure their sequence similarities, which does not quite fit our use case. The main problem is that we require an algorithm that does not treat all matches and mismatches in the same way. This stems from the fact that some amino acid types have a more similar appearance in cryo-EM maps than others, which leads to some mismatches of the U-Net being more likely than others. To analyze the relative frequency of a certain match of predicted and true amino acid types, we applied the U-Net to 200 different maps and compared the predicted amino acid types with the actual types from the deposited model structures. The heatmap depicting this analysis is shown in Fig. 6. As expected, the most frequent matches are those of the same predicted and true amino acid types. However, we can also see that the U-Net often mixes up some types (e.g., ALA and SER) and struggles more with other types (e.g., CYS).

To incorporate the U-Net prediction behavior described in the previous section into the alignment algorithm, we defined a reward function r which returns a score denoting how valuable a certain match of predicted type p and true type t is. With f(p,t) defined as the relative frequency of a match, we constructed the reward function shown in Eq. **1**. The constant 100 as a multiplier is used to balance the match rewards with gap penalties described in the next section, and was chosen based on multiple rounds of testing. The 0.05 constant was chosen as this represents the likelihood of a correct match if we would choose the amino acid type randomly, since there are 20 different types of amino acids. The score is zero if the relative frequency equals this random likelihood.

$$r\left(t_{p},t_{t}\right)=100\times \left(f\left(p,t\right)-0.05\right).$$ [1]

In addition to the match reward, our algorithm also requires a gap penalty. A gap represents a skipped amino acid in either the predicted or true sequence. This penalty, however, cannot simply be a static value, as not all gaps are the same. For example, gaps in the beginning of a sequence before any matches were made should not result in any penalties, as we only match short intervals of the predicted sequence, meaning it is highly unlikely that they align at the first amino acid of the true sequence. Additionally, the number of consecutive gaps is important. Cases where DeepTracer misses an amino acid or predicts an extra amino acid appear relatively frequent, meaning that a single gap is not unlikely. However, two missed amino acids in a row is very uncommon, and three gaps in a row virtually never happens. Therefore, we must define our penalty function p such that it takes the number of consecutive gaps g into account. Let i be the index of the amino acid that is not skipped. Then we can define p as shown in Eq. **2**. The constants 20 and 30 were chosen based on test runs to create a good balance with the rewards function.

$$p\left(g,i\right)=\left\{\begin{array}{cc} 0,\; & \text{if}\;i=0\\ \infty ,\; & \text{if}\;g\geq 3\\ 20+\left(g\times 30\right),\; & \text{otherwise} \end{array}\right..$$ [2]

Since we have defined a reward and penalty function, we can find the ideal alignment by maximizing the sum of all rewards and penalties using a dynamic algorithm. To do so, we defined a recursive equation which calculates the optimal solution based on an index i, which points to the current amino acid in the true sequence; an index j, which points to the current amino acid in the predicted sequence; and g, which counts the number of previous consecutive gaps. With t and p as the true and predicted sequence, we defined this function as shown in Eq. **3**. To efficiently find the solution, we applied the dynamic programming “bottom up” approach (27).

$$\text{OPT}\left(i,j,g\right)=\left\{\begin{array}{c} 0,\;\\ \;\text{if}\;i=0\;\text{or}\;j=0\;\text{or}\;g\geq 3\;\\ \text{max}\left\{\text{OPT}\left(i-1,j-1,0\right)+r\left(t_{i},p_{j}\right),\right.\;\\ \;\text{OPT}\left(i,j-1,g+1\right)+p\left(g,i\right),\;\\ \;\left.\;\text{OPT}\left(i-1,j,g+1\right)+p\left(g,j\right)\right\},\;\\ \;\text{otherwise}\; \end{array}\right..$$ [3]

### Carbon, Nitrogen, and Oxygen Determination.

So far, the determined residues consist solely of Cα atoms. A complete protein backbone also consists of carbon, nitrogen, and oxygen atoms. Previous research has introduced various methods for reconstruction of a protein backbone from a reduced representation, such as one that contains only Cα atoms (28). Instead of employing these theoretical methods, we chose to implement our own backbone reconstruction method to make use of the information captured from the 3D cryo-EM maps. This section presents our all-atom backbone reconstruction method. This is necessary for the next step in the pipeline, resolving the side-chain atoms.

**Fig. 7.**
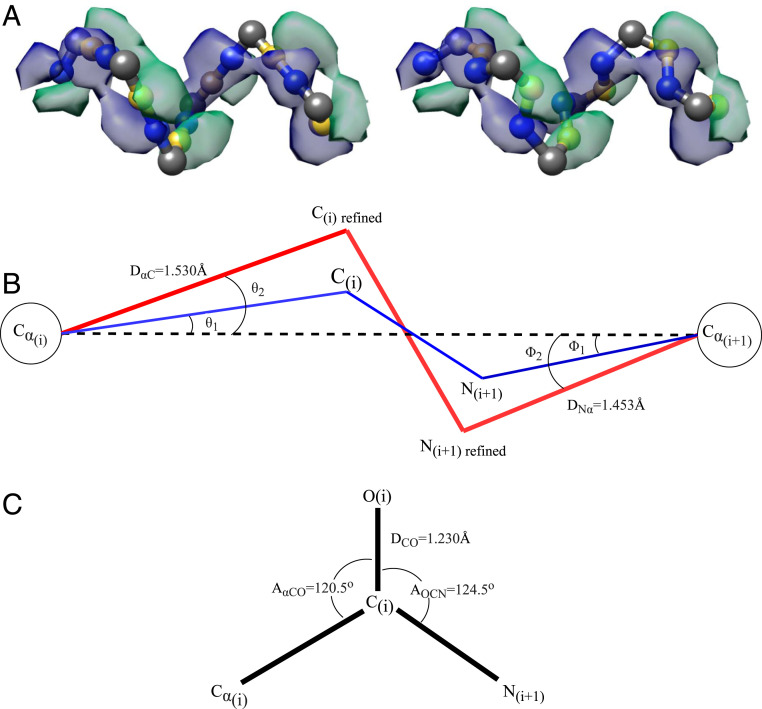
Carbon, nitrogen, and oxygen determination. (*A*) Initial positioning of carbon (yellow) and nitrogen (blue) atoms in between the Cα atoms (gray) on *Left* and their initial refined positioning, which fits the U-Net prediction of carbon atoms (green volume) and nitrogen atoms (blue volume), on *Right*. (*B*) The positions of carbon and nitrogen atoms are refined further by forcing bond angles into their well-known values. The blue lines represent the bonds from the initial refinement. The red lines represent the bonds from the final refinement. (*C*) Position of oxygen atom in the carbonyl group by definition.

In addition to Cα prediction, the U-Net also provides information about carbon and nitrogen atoms in the confidence map predicted by the U-Net. We can use this information in combination with the previously determined Cα atom positions to place the carbon and nitrogen atoms. Between the Cα atoms of two connected amino acids, there is always a nitrogen and a carbon atom. Therefore, we can guess the initial position of these atoms by calculating the vector from one Cα atom to the other and then placing the nitrogen and carbon atoms at one-third and two-thirds of the distance of this vector. To refine these initial positions, we calculated the center of mass around them in the carbon and nitrogen confidence maps. In Fig. 7*A*, we can see an example for the initial and refined placement of the carbon and nitrogen atoms.

After the initial refinement, we can further refine the positions of the carbon and nitrogen atoms by applying well-known molecular mechanics of a peptide chain. We made several assumptions about the positions of carbon, nitrogen, and oxygen atoms relative to the Cα atoms, as seen in Fig. 7*B*. First, we assumed the planar peptide geometry in which the Cα atom and carbon atom in the carbonyl group of an amino acid are in the same plane as the next amino acid’s nitrogen and Cα atom (29). Second, we constructed a virtual bond between the neighboring Cα atoms. The angles between this bond and the C$\alpha_{\left(i\right)}$–$C_{\left(i\right)}$ bond ($\theta_{2}$) and between this bond and the C$\alpha_{\left(i+1\right)}$–$N_{\left(i+1\right)}$ bond ($\phi_{2}$) are 20.9° and 14.9°, respectively (29). Third, the peptide bonds in a protein are in the stable trans configuration (30).

To refine the position of the carbon atoms, we relied on the previous refinement. Let us call the unit vector pointing from C$\alpha_{\left(i\right)}$ to $C_{\left(i\right)refined}\;v_{1}$, the unit vector pointing from C$\alpha_{\left(i\right)}$ to $C_{\left(i\right)}\;v_{2}$, and the unit vector pointing from C$\alpha_{\left(i\right)}$ to C$\alpha_{\left(i+1\right)}\;v_{3}$.

$$\begin{array}{l} v_{1}\;=\;<a_{1},a_{2},a_{3}>\\ v_{2}\;=\;<b_{1},b_{2},b_{3}>\\ v_{3}\;=\;<c_{1},c_{2},c_{3}>. \end{array}$$ [4]

The goal is to solve for the components of $v_{1}$. Due to the planar peptide geometry, $v_{1}$, $v_{2}$, and $v_{3}$ exist in the same plane. Thus, their triple product equals zero.

$$v_{1}\times \left(v_{2}\cdot v_{3}\right)=0$$ [5]

$$\begin{array}{l} or\\ a_{1}\left(b_{2}c_{3}-b_{3}c_{2}\right)-a_{2}\left(b_{1}c_{3}-b_{3}c_{1}\right)+a_{3}\left(b_{1}c_{2}-b_{2}c_{1}\right)=0. \end{array}$$ [6]

From this relation and the cross-product of $v_{1}$ and $v_{2}$, and that of $v_{2}$, $v_{3}$, we can construct a system of equations,

$$\begin{array}{l} \left\{\begin{array}{c} a_{1}b_{1}+a_{2}b_{2}+a_{3}b_{3}=cos\left(\theta_{2}-\theta_{1}\right)\;\\ a_{1}c_{1}+a_{2}c_{2}+a_{3}c_{3}=cos\left(\theta_{2}\right)\;\\ a_{1}\left(b_{2}c_{3}-b_{3}c_{2}\right)-a_{2}\left(b_{1}c_{3}-b_{3}c_{1}\right)+a_{3}\left(b_{1}c_{2}-b_{2}c_{1}\right)=0\; \end{array}\right.. \end{array}$$ [7]

Solving this system of equations yields $a_{1}$, $a_{2}$ and $a_{3}$. Next, the vector $v_{1}$ is scaled appropriately to resolve the new position of the carbon atom. The position of the nitrogen atom is refined in a similar manner.

To determine the location of the oxygen atom in the carbonyl group, we assumed the coplanar relationship between the oxygen, Cα, carbon, and nitrogen atom (29), and that the angles $A_{\alpha CO}$ and $A_{OCN}$ (Fig. 7*C*) are approximately identical. We then derived a unit vector pointing in the direction of the C–O bond and scaled it with the C–O bond length to get the position of the oxygen atom.

### Side-Chain Prediction.

The final step of DeepTracer is the side-chain prediction. Its goal is to position the side-chain atoms of each amino acid based on its type and backbone structure. This is done by using SCWRL4 (31), a tool developed by the Dunbrack laboratory, which predicts side-chain atoms for structures that have a complete backbone and amino acid types set. The tool is integrated in the pipeline of DeepTracer and runs fully automatically as well. It also performs a collision detection to ensure that side chains of different residues do not overlap. In *SI Appendix*, Fig. S7, we can see an example of an α-helix after the side-chain prediction step.

## Results

We evaluated the effectiveness of DeepTracer by applying it to multiple test datasets of experimental cryo-EM maps, most of which depict multichain complexes. As a point of comparison, we used results generated by Phenix’s map-to-model function. Further comparative tests with Rosetta and MAINMAST method can be found in *SI Appendix*.

### Metrics.

To ensure the objectivity of the comparison with the existing Phenix method, we used the phenix.chain_comparison tool (32), which is available at no cost as part of the Phenix software suite. This tool compares two models by finding a one-to-one match between their residues based on Cα positions. For two residues to match, they cannot be farther apart from each other than 3 Å. Based on this matching, several metrics are calculated. The first metric is the rmsd, which expresses the average distance between Cα atoms of matched residues. Second, the coverage is expressed using the matching percentage. This value represents the proportion of residues from the deposited model which have a matching interpreted residue, and is calculated by dividing the number of matches by the total number of residues. Third, to evaluate how well the amino acid types were predicted, the chain_comparison tool calculates the sequence matching percentage, which denotes the percentage of matched residues that have the same amino acid type. Lastly, to get a sense of how similar residues are connected, the mean length of matched segments is calculated where consecutive matches are connected in both models. Besides the metrics calculated by the phenix.chain_comparison tool, we also apply the LGA (Local–Global Alignment) algorithm, which aligns two models and computes the GDC (Global Distance Calculation) score. This score measures the similarity of two structures based on all atoms (including side chains) on a range of 0 to 100 with 100 being a perfect match (33, 34). We applied it on the most important dataset of severe acute respiratory syndrome coronavirus 2 SARS-CoV-2 cryo-EM maps due to the high manual and computational effort involved in the calculation of this metric.

### Phenix Benchmark Test.

**Fig. 8.**
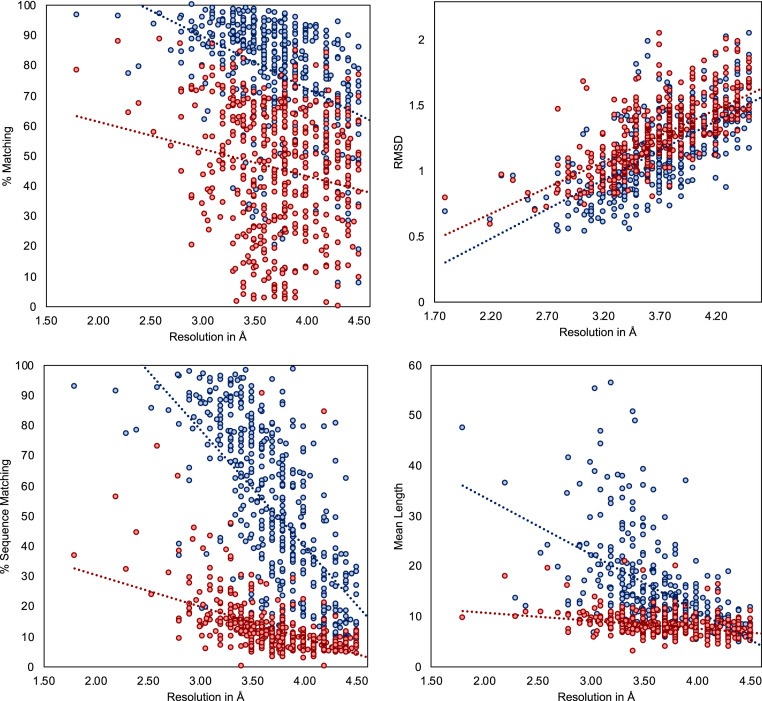
Evaluation results for set of 476 experimental cryo-EM maps. Evaluation of determined models from DeepTracer (blue) and Phenix (red) for 476 cryo-EM maps. The dotted lines represent the trend for each method. DeepTracer outperformed Phenix in all four metrics. Precise data can be found in *SI Appendix*, Table S3.

We applied DeepTracer on a set of 476 cryo-EM maps assembled by the authors of Phenix’s map-to-model method (11) and compared the determined models against the ones published on Phenix’s website (35) (Fig. 8). We can see that DeepTracer achieves better results than the Phenix method for every metric calculated by the phenix.chain_comparison tool. The matching percentage of deposited model residues is, on average, 76.93% compared to 45.65% with Phenix, representing an improvement of over 30%. DeepTracer achieved a matching percentage above 70% for almost all cryo-EM maps, except a few outliers. The average rmsd value of DeepTracer (1.29) is 0.11 higher than Phenix’s (1.18). We can see that the distribution of the rmsd values of DeepTracer follows a similar pattern as Phenix, with a strong correlation between rmsd and the resolution of the map. The most significant improvements of DeepTracer were measured for the sequence matching which expresses the percentage of matched residues in the determined and deposited model that have the same amino acid type. For this metric, DeepTracer achieved 49.83%, which is more than 4 times higher than the 12.29% of the Phenix method. Although 49.83% is still fairly low, the distribution of the values shows that there is a steep improvement of the sequence matching with more-accurate maps. Two factors contribute to this trend. First, side-chain atoms, which determine the amino acid type, are only visible in very high-resolution maps, making it almost impossible to accurately predict the amino acid type for lower resolutions. Second, the amino acid type mapping of every segment can be either correct or incorrect. It means that either all amino acid types will be correct for this segment or, in the case of an incorrect mapping, the amino acid types are entirely random. This amplifies the steep incline in accuracy for higher-resolution maps. Third, for the last evaluated metric, the mean length of matched segments improved from 8.16 with Phenix to 14.05 with DeepTracer. While this number is influenced by several factors, including the average length of connected segments in the deposited model structure, this is an indicator that DeepTracer connects residues better than the Phenix method.

**Fig. 9.**
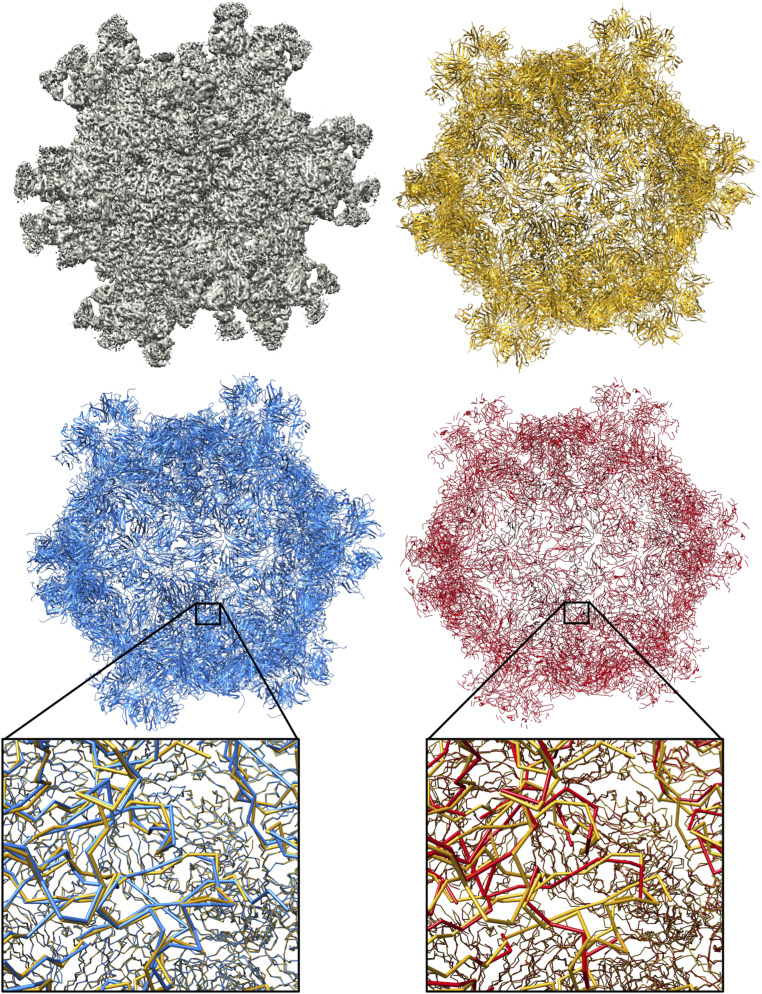
Results of EMD-6757 map. Models built by DeepTracer (blue) and Phenix (red) next to PDB ID code 5XS7 deposited model structure (yellow) for EMD-6757 map.

**Fig. 10.**
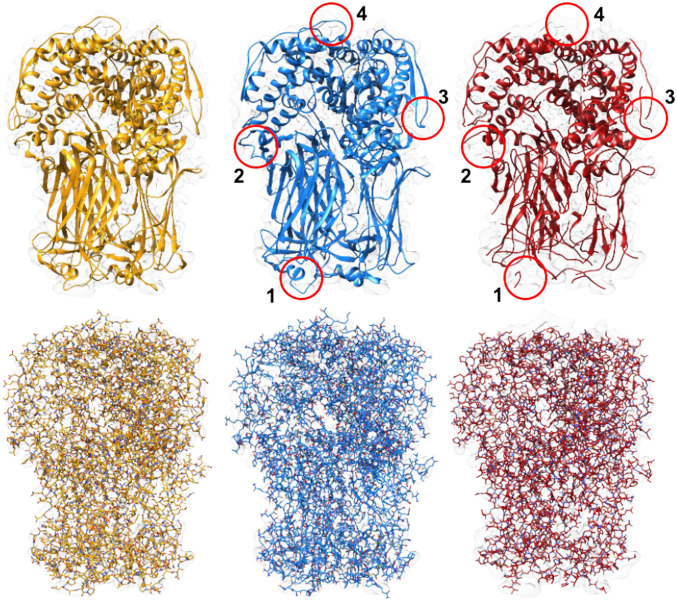
Results of EMD-6272 map. Models built by DeepTracer (blue) and Phenix (red) compared to PDB ID code 3J9S deposited model structure (yellow) for EMD-6272 map. *Top* shows structures in ribbon view, and *Bottom* shows structures in all-atom view. Areas where DeepTracer correctly predicted amino acids that Phenix missed are highlighted by the four red circles.

Figs. 9 and 10 show multichain complexes modeled by DeepTracer and Phenix compared to the deposited model structure. In both figures, we can note that DeepTracer’s model is more complete. In Fig. 9, we can particularly see the greater coverage and more precise placement of the residues in direct comparison with the ground truth. Fig. 10 shows well that, even though Phenix determined most of the residues correctly, DeepTracer connected the residues better, creating a less fragmented model.

### Coronavirus-Related Results.

In the search for an effective COVID-19 vaccine and medicine, structural information about the viral protein is crucial. Therefore, we applied DeepTracer on a set of coronavirus-related cryo-EM maps to demonstrate how it can aid researchers in obtaining such structural information. To create a point of comparison, we applied Phenix on the same set of cryo-EM maps. The dataset was aggregated by the EMDataResource and contained 62 high-resolution cryo-EM maps, 52 of which have a deposited model Protein Data Bank (PDB) structure (36). The dataset as well as the determined models will be actively updated at DeepTracer’s website as more and more data are deposited to EMDR (EMDataResource).

**Fig. 11.**
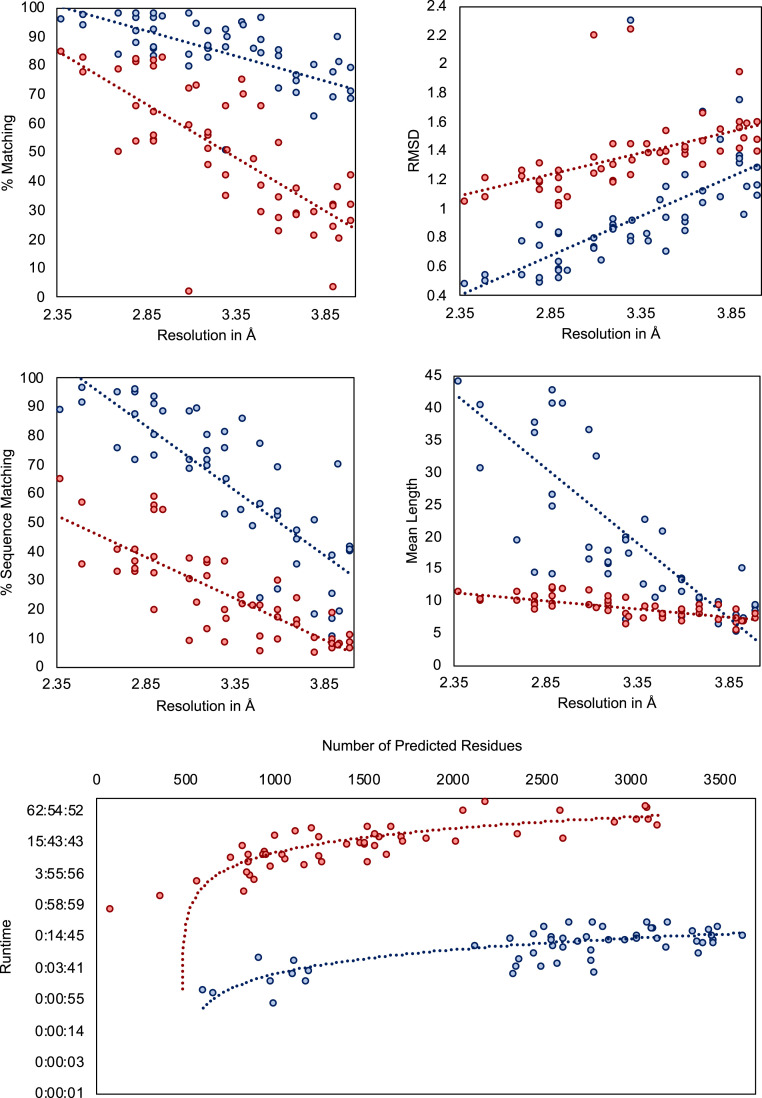
Results for coronavirus-related cryo-EM maps. Evaluation of models built by DeepTracer (blue) and Phenix (red) for 52 coronavirus-related high-resolution cryo-EM maps. The dotted lines represent the trend for each method. Computation times are shown on a logarithmic scale. Precise data can be found in *SI Appendix*, Table S2.

The scatter plots in Fig. 11 show the evaluation results for the metrics calculated by Phenix’s chain_comparison tool, for the 52 coronavirus-related cryo-EM maps that have a deposited model structure. The average percentage of matched model residues is 84% for DeepTracer and 49.8% for Phenix. This means that, on average, around 34% more residues were correctly placed by DeepTracer than by Phenix. The rmsd metric calculated an average value of 1.37 Å for Phenix compared to 0.93 Å with DeepTracer. Thus, DeepTracer not only determines more residues correctly than Phenix, but the correctly determined residues were also closer to the residues of the deposited model by around 0.4 Å. For the sequence matching results, Phenix scored 24.95%, while DeepTracer achieved a sequence matching percentage of 63.08%. Finally, the mean length of consecutively matched residues in the modeled and deposited structure increased from 8.9 with Phenix to 20 with DeepTracer.

The SARS-CoV-2 results from Table 1 show a pattern similar to the results of all coronavirus-related maps. DeepTracer outperformed Phenix in every metric with the most significant differences in the matching percentage and sequence matching. Additionally, the DeepTracer achieved a GDC score almost 3 times that of the Phenix method.

**Table 1.** Comparison of DeepTracer (DT) and Phenix (P) for SARS-CoV-2 dataset

			Percent matching		rmsd		Percent sequence ID		GDC	
lr0.5em)4-5lr0.5em)6-7lr0.5em)8-9l)10-11 EMDB	PDB	Residues	DT	P	DT	P	DT	P	DT	P
21375	6vsb	2905	84.90	48.60	1.14	1.40	45.90	20.90	17.88	5.39
21452	6vxx	2916	91.40	53.80	0.96	1.18	61.30	40.00	—	—
30039	6m17	3072	80.30	53.10	1.72	1.72	69.80	54.60	11.84	8.31
30127	6m71	1077	91.70	54.20	1.02	1.20	58.60	16.60	20.74	8.89
30178	7btf	1227	94.90	81.00	0.83	1.09	85.80	51.80	65.57	23.06
30209	7bv1	1102	87.60	67.00	0.84	1.29	87.50	30.50	55.62	18.15
30210	7bv2	1006	92.40	78.20	0.78	1.08	88.90	53.70	40.90	13.32
Average			89.03	62.27	1.04	1.28	71.11	38.30	35.42	12.85

GDC score could not be calculated for the EMD-21452 map, as the LGA web service could not process the modeled structures due to their size. EMDB, Electron Microscopy Data Bank.

**Fig. 12.**
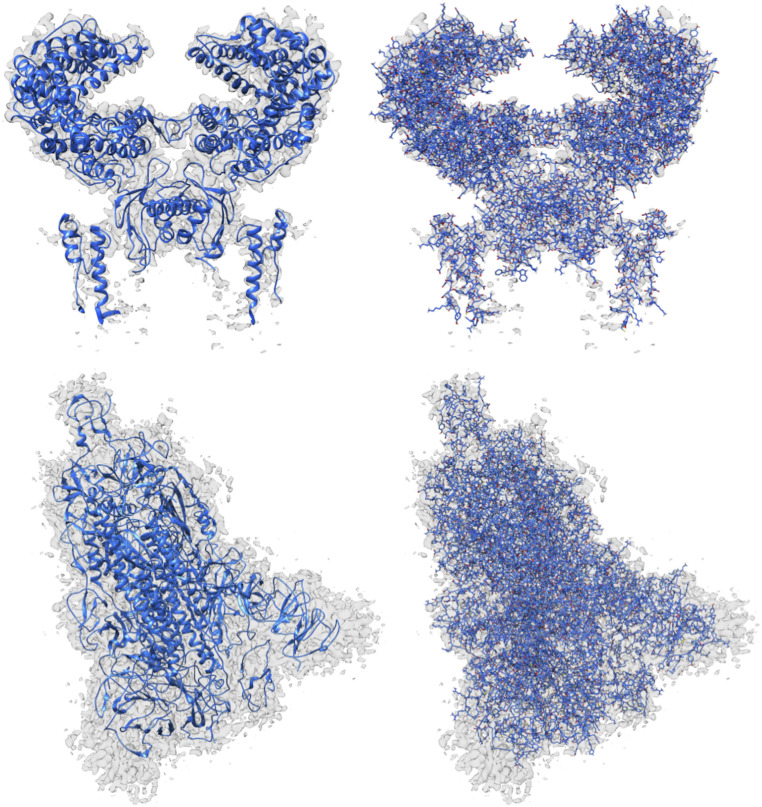
Models built from SARS-CoV-2 cryo-EM maps, which do not have deposited model structures in the EMDR. DeepTracer model for the EMD-30044 map (*Top*) showing a human receptor ACE2 to which spike proteins of the SARS-CoV-2 virus bind and (*Bottom*) the EMD-21374 depicting a SARS-CoV-2 spike glycoprotein. No model structure has been deposited to the EMDataResource for the cryo-EM maps as of the date this paper is announced.

In Fig. 12, we can see the structures modeled by DeepTracer for the EMD-30044 map, which captures the human receptor angiotensin-converting enzyme 2 (ACE2) to which the spike protein of the SARS-CoV-2 virus binds (8) and the EMD-21374 map of a SARS-CoV-2 spike glycoprotein. No model structure has been deposited to the EMDR for either map as of the date this paper is announced. This represents an ideal opportunity to showcase the potential of DeepTracer. Without any other parameters or manual processing steps, DeepTracer can determine detailed models based on the cryo-EM maps. Researchers can use these models to develop therapeutics targeting the binding process between the spike protein and the human enzyme.

### Computation Time.

A major bottleneck of existing methods is their computational complexity, which renders them unable to model larger protein complexes. Thus, we conducted an analysis of DeepTracer’s computational time versus Phenix’s. The result is shown in Fig. 11. The tests were executed on a machine with an Nvidia GeForce GTX 1080 Ti graphics processing unit (GPU), eight processors, and 62 GB of memory. Although a comparison with the Phenix method is not entirely fair, as Phenix does not take advantage of the machine’s GPU, this comparison provides a glimpse of the possibility that DeepTracer can achieve. We observed that Phenix took about 45 min to process a map containing 79 residues, while DeepTracer processed a map containing 2,798 residues in only 26 min. Furthermore, the largest cryo-EM map (EMD-9891) that DeepTracer was tested on required around 14 min to complete, whereas Phenix’s processing time for this map was over 60 h. DeepTracer is able to exploit the processing power of the GPU, which is becoming a staple on modern computing systems, to increase the throughput of scientific discovery. DeepTracer can model even very large protein complexes in a matter of hours. As an example, it traced around 60,000 residues for the EMD-9829 map within only 2 h.

## Discussion

In this paper, we present DeepTracer, a fully automatic tool that determines the all-atom structures of protein complexes based on their cryo-EM maps, using a tailored deep convolutional neural network and a set of computational methods. We applied this software on a set of coronavirus-related cryo-EM maps and compared the results to Phenix, the state of the art cryo-EM model determination method (11). We found that DeepTracer correctly placed, on average, around 30% more residues than Phenix with an average rmsd improvement of 0.11 Å, from 1.29 Å to 1.18 Å. We also applied DeepTracer on a dataset of coronavirus-related cryo-EM maps and calculated a coverage of 84% compared to 49.8% with Phenix and an average rmsd value of 0.93 Å for DeepTracer and 1.37 Å for Phenix. Detailed description and discussion can be found in *SI Appendix*. Furthermore, we compared DeepTracer with Rosetta and MAINMAST on a previously published set of nine cryo-EM maps and observed significant rmsd improvements in comparison with Rosetta, from 1.37 Å to 0.85 Å, and a much more complete model compared to MAINMAST, with a coverage increase of 57%, from 36.4% to 93.4%. Detailed description and discussion can be found in *SI Appendix*. These results represent a significant accuracy boost, resulting in more-complete protein structures. Particularly, for large protein complexes, DeepTracer built models much faster than other methods, tracing tens of thousands of residues with million of atoms within only a few hours. We achieved the results without any manual preprocessing steps, such as zoning or cutting of the cryo-EM map using a deposited model structure. This means we can determine models without any prior knowledge about the cryo-EM map, and the users do not need to tune any parameters in order to obtain an accurate structure.

All-atom modeling from experimental cryo-EM data is challenging. Due to the experimental noises from various aspects of upstream cryo-EM single-particle analysis workflow (e.g., sample preparation, image acquisition, and processing), the resulting atomic structures from DeepTracer may contain unavoidable geometric issues, local fit-to-map issues, occasionally misplaced side chains, and occasional tracing/connectivity errors. DeepTracer could be pipelined with other refinement and validation tools, such as molecular dynamics flexible fitting programs, to perform additional rebuilding and rerefinement (37, 38). Researchers reported that, with multiple rounds of rebuilding (in both the globally sharpened and local resolution-filtered maps), real-space refinement in Phenix (39) using secondary structure, rotamer, and Ramachandran restraints, and candidate model validation using MolProbity in Phenix (40), one could yield better final models (41).

As the cryo-EM technology becomes more readily available, the number of captured cryo-EM maps, especially larger protein complexes, is rising rapidly. DeepTracer allows for a greater throughput of cryo-EM, as it can automatically and accurately infer structural information from cryo-EM maps of macromolecule. This outcome ultimately accelerates the scientific discovery process, which is particularly urgent today, given the ongoing coronavirus pandemic. Coronavirus-related cryo-EM maps are deposited to the EMDR on a daily basis. Our efficient and automated method to model these maps is an important tool for researchers to resolve the structural information of the virus-related macromolecules.

## Supplementary Material

Supplementary File

## Data Availability

Application programming interfaces have been deposited on the DeepTracer website (https://deeptracer.uw.edu). All study data are included in the article and *SI Appendix*.
