# Supplementary material for: DeepTracer for fast de novo cryo-EM protein structure modeling and special studies on CoV-related complexes
Source: Proc Natl Acad Sci U S A. 2020 Dec 23;118(2):e2017525118. doi: 10.1073/pnas.2017525118 (PMC7812826; doi:10.1073/pnas.2017525118)
Supplement: Supplementary File [file pnas.2017525118.sapp.pdf]

SI Appendix: DeepTracer for Fast De Novo Cryo-EM Protein Structure Modeling and Special Studies on CoV-related Complexes

Jonas Pfab[1], Nhut Minh Phan[1], and Dong Si[1,*]

[1]Division of Computing and Software Systems, University of Washington, Bothell, WA, 98011, USA

[*]Corresponding author: Dong Si, dongsi@uw.edu

December 7, 2020

S1 Pre-Processing

The goal of the pre-processing steps is to prepare the cryo-EM maps for the neural network. These steps are crucial as cryo-EM maps can differ significantly in terms of shape, quality, resolution, and more. Therefore, we need to process the maps and convert them into a consistent format such that the neural network can understand connections across different maps. The pre-processing steps that achieve this are examined in Sections S1.1, S1.2, and S1.3.

S1.1 Data Grid Resampling

The first pre-processing step is to standardize the voxel size of all cryo-EM maps. The grid storing the volume data of the map has an associated voxel size, which determines the size of a single grid element or voxel in Angstrom. Without standardizing this voxel size to a fixed value the neural network could not draw conclusions about how far two voxels are from each other, making it difficult to predict the location of any amino acids. Therefore, this step ensures that each maps has a voxel size of exactly 0.5Å. The value 0.5 was chosen based on several rounds of testing as a trade-off between prediction precision and memory usage of the resulting grids.

To set the voxel size of a cryo-EM map to 0.5Å, we cannot simply change the meta data of the map. We had to resample the volume data onto a new grid in which each voxel represents 0.5Å. An example of a resampling process from an origin grid to a grid with half the voxel size is shown in Figure S1. Here, the shape of the volume remains the same, however, we require eight times the number of voxels to represent it. To realize the resampling step, DeepTracer utilizes UCSF Chimera [1]. First, it creates a new grid of the same size in Angstrom as the original map, but with a voxel size of 0.5Å. Then, it uses Chimera's resampling command to resample the original map onto the newly created grid.

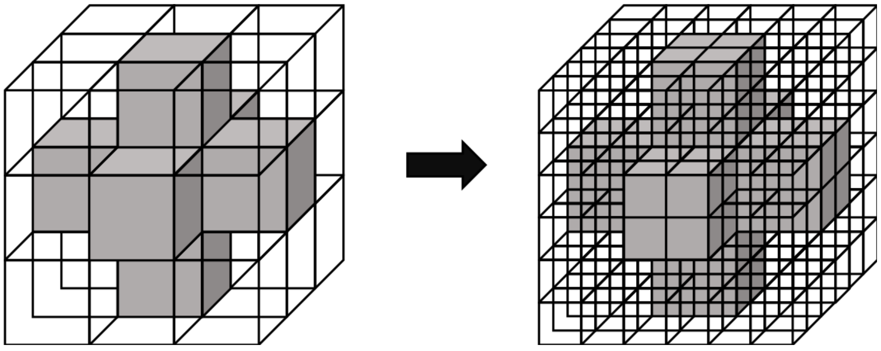

Figure S1: Data grid resampling. Visualization of the resampling process from an origin grid onto a grid with half the voxel size.

S1.2 Density Value Normalization

The absolute value of a voxel in itself contains little information. We have to rely on the density values of other voxels to make conclusions about the protein structure. Consequently, we can normalize the density values without risking information loss. The normalization process makes sure that the range of density values is identical for all maps. In the case of experimental maps, this range can initially differ substantially with some maps contain values from -0.1 to 0.1 and other maps ranging from -10 to 20.

To normalize values, we can usually divide each value by the overall highest value. However, this process is problematic for some cryo-EM maps as there are outlier density values, which have values that are much higher than all other values. If we were to divide all other values using these outlier, all other density values would end up being close to zero. Therefore, we used the 95th percentile of the density values to divide all other values with. Afterwards, we simply set the few values that are greater than 1 to 1. Additionally, we set all values below 0 to 0 as they contain no valuable information for our use case. This leaves us with a range from 0 to 1, which contains all density values. An example of the density value histograms before and after the normalization step can be seen in Figure S2.

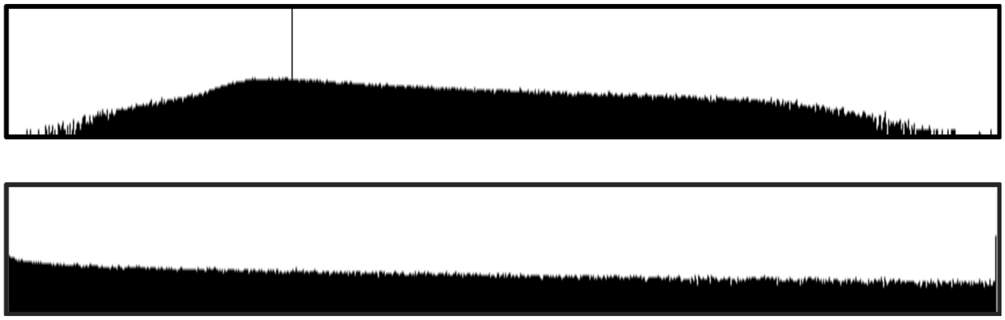

Figure S2: Density value normalization. Histograms of the 6272 cryo-EM map depicting the relative frequency of density values before (top) and after (bottom) normalization. The vertical black line in the upper histogram indicates the value 0 and is not visible in the lower one as there are no density values below 0 anymore.

S1.3 Grid Division

The input layer of the model takes a cryo-EM map to make a prediction. We have to make sure that the dimensions of the volume data grid of the map are identical to those of the input layer of the model to avoid mismatching errors. However, the dimensions of the grid vary from map to map, demanding modification of the grid to match its dimensions of the input layer. Unfortunately, we cannot simply scale the map for it to fit the input layer as this would change the size of each voxel in Angstrom, which has to remain 0.5 as mentioned in Section S1.1. Therefore, we divided the grid into multiple sub grids each the size of the input layer of the deep learning model.

We divided the volume data grid into sub grids of size 64^3. The number 64 was chosen as it creates a relatively small input layer that is still broad enough for the deep learning model to detect larger patterns, such as secondary structure elements. Dividing the grid, however, can aggravate predictions in areas close to the border of sub grids as relevant information from neighboring voxels might be cut off. Therefore, we introduce a core grid of size 50^3 in the center of each sub grid. Although each sub grid has a size of 64^3, we only used the predictions from the inner core grid. Consequently, when dividing the grid we overlapped the 64^3 sub grids such that core grids of all sub grids cover the entire original grid without overlap. An example of such a division for a two-dimensional grid can be seen in Figure S3.

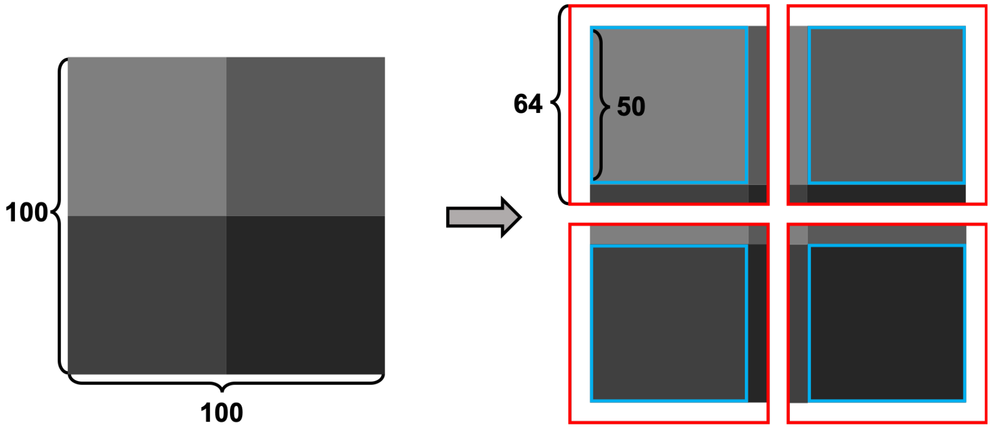

Figure S3: Grid division into sub-grids. Visualization of the division of a 100^2 grid into four grids each with a dimension of 64^2 and a core size of 50^2. The red lines indicate the complete sub grids while the blue lines show their cores.

S2 Comparison with MAINMAST and Rosetta

In addition to Phenix, MAINMAST and Rosetta are two further established cryo-EM prediction methods. We conducted a brief analysis of their performances compared to DeepTracer based on a test set of nine cryo-EM maps taken from the previous papers [2, 3]. Note that the cryo-EM maps were cropped such that they captured only a single protein chain. This cropping was necessary as both methods can only perform single-chain predictions. To evaluate predictions we utilize Phenix's `chain_comparison` tool. The results of this analysis can be seen in Table S1. We can note that DeepTracer outperforms Rosetta in all four metrics with particularly significant improvements in the percentage of matched residues as well as false-positive predictions. Compared to the MAINMAST method DeepTracer performed worse in three of the four metrics. However, predictions of DeepTracer were much more complete with an average matching percentage of 93.4% compared to only 36.4% with MAINMAST. That means that MAINMAST correctly predicted only around 1/3 of the protein structure.

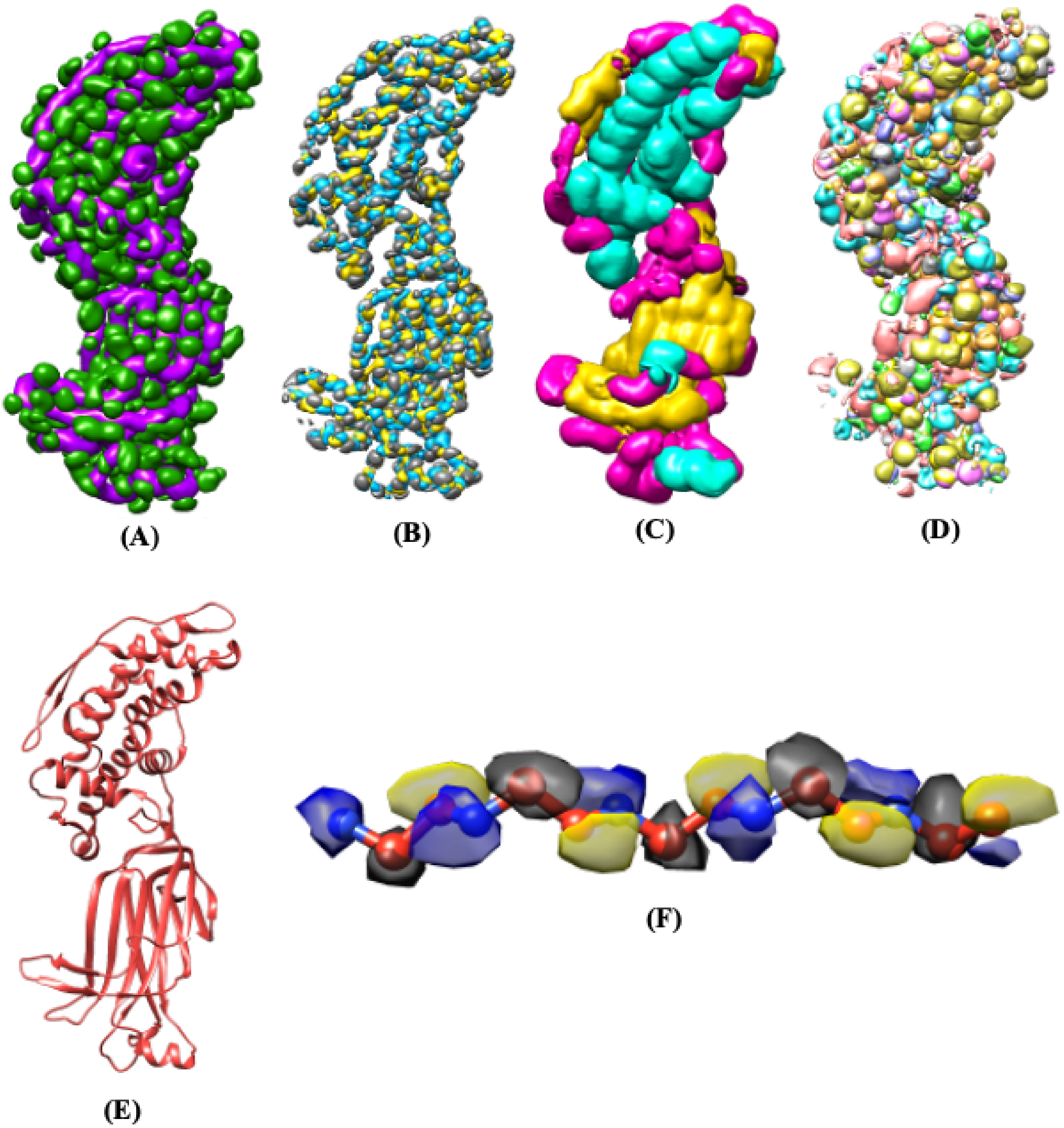

Figure S4: Neural network predictions. Raw prediction of the U-Net deep learning model for the 6272 cryo-EM map. (A) Backbone prediction with backbone in purple and side-chains in green. (B) Atoms prediction with Cα atoms in gray, C atoms in yellow, and N atoms in blue. (C) Secondary structure prediction with α-helices in turquoise, β-sheets in yellow and loops in pink. (D) Amino acid type prediction with a different color for each type. Here, we can see that some amino acids are hard to discern. This comes from the fact that there are 20 different amino acids that the neural network has to predict and that some amino acids could look very similar in an experimental cryo-EM map. (E) Solved structure (PDB-3J9S) of cryo-EM map. (F) Atoms prediction segment next to solved structure.

Table S1: Comparison of DeepTracer with MAINMAST and Rosetta on a dataset of 9 cryo-EM maps.

Method	Protein	% Matching	RMSD	% Seq Matching	% FP
DeepTracer	BPP1	93.30	0.82	66.20	2.56
	FrhB	98.20	0.67	68.40	3.17
	T20S	86.40	1.19	33.50	4.55
	VP6	93.20	0.98	48.10	2.38
	TRPV1	87.70	0.85	71.30	3.20
	FrhA	97.90	0.60	98.10	1.31
	FrhG	96.10	0.86	90.40	5.63
	STIV	90.40	0.88	70.10	0.32
	TMV	97.40	0.84	58.30	3.21
	**Avg.**	**93.40**	**0.85**	**67.16**	**2.93**
Rosetta	BPP1	73.10	1.51	58.20	28.44
	FrhB	90.00	1.07	93.30	10.68
	T20S	77.40	1.61	62.60	24.89
	VP6	72.00	1.37	48.60	28.72
	TRPV1	84.20	1.22	69.70	18.10
	FrhA	92.50	0.90	93.30	8.29
	FrhG	63.20	1.79	37.50	41.23
	STIV	47.70	1.65	46.30	52.75
	TMV	91.00	1.20	88.70	10.97
	**Avg.**	**76.79**	**1.37**	**66.47**	**24.89**
MAINMAST	BPP1	17.40	0.68	100.00	0.00
	FrhB	59.60	0.77	98.80	2.34
	T20S	23.10	1.01	100.00	1.96
	VP6	26.20	0.73	99.00	0.00
	TRPV1	33.20	0.72	99.00	0.00
	FrhA	57.70	0.62	97.70	0.89
	FrhG	31.10	0.72	100.00	1.39
	STIV	14.80	0.68	100.00	0.00
	TMV	64.50	0.78	98.00	0.99
	**Avg.**	**36.40**	**0.75**	**99.17**	**0.84**

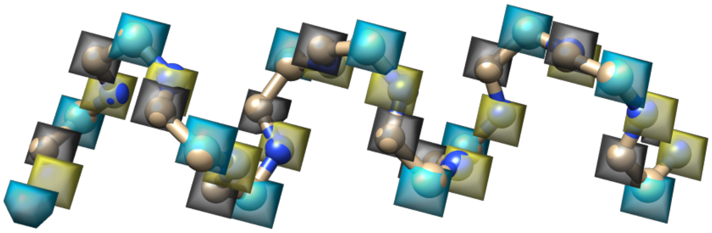

Figure S5: Atom mask. Portion of the atom mask containing backbone atoms for part of a helix from the PDB-6NQ1 structure. The gray labels indicate carbon alpha atoms, the blue labels carbon atoms, and the yellow labels nitrogen atoms.

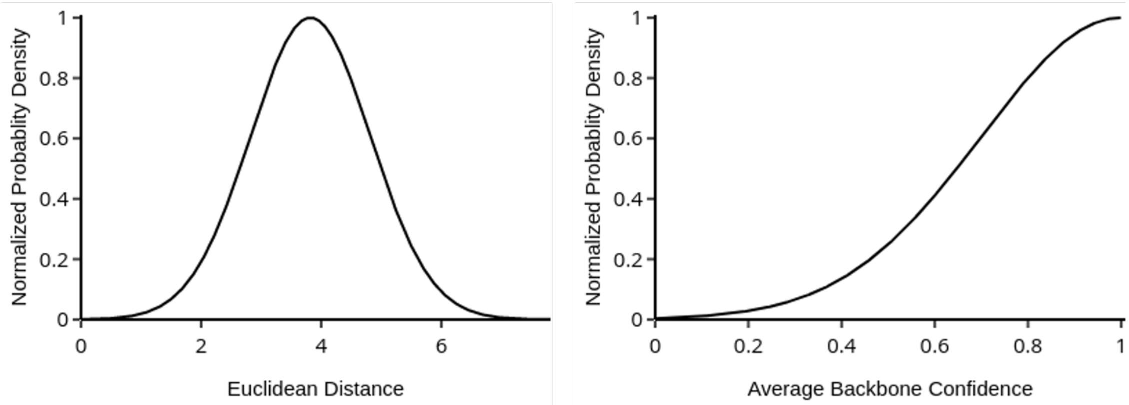

Figure S6: Probability density function for connection confidence. Normalized probability density function used to calculate confidence score for the euclidean distance and average backbone confidence between two Cα atoms. This is used to express a distance between two atoms for the traveling salesman algorithm tracing the backbone.

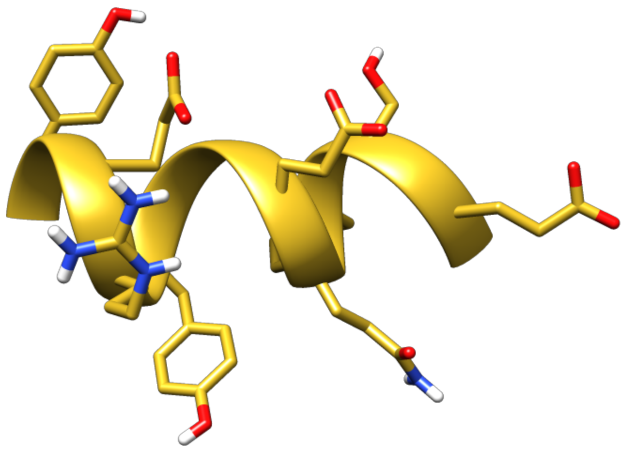

Figure S7: Predicted side chains for an extract of an α-helix. Backbone atoms are displayed in the ribbon view and side chain atoms in atom view.

Table S2: Precise prediction results for coronavirus-related maps. Evaluation of prediction results from DeepTracer and Phenix for 52 coronavirus-related high-resolution cryo-EM maps.

	**DeepTracer**				**Phenix**			
EMDB ID	% Matching	RMSD	% Seq Matching	Mean Length	% Matching	RMSD	% Seq Matching	Mean Length
0401	84.30	0.93	55.90	11.70	29.00	1.39	9.90	7.70
0402	83.10	0.90	51.80	13.40	34.40	1.40	19.30	7.60
0520	97.70	0.73	87.70	36.60	71.80	1.24	37.10	9.20
0521	88.80	1.15	23.30	7.70	38.20	1.53	5.10	7.20
0557	96.10	0.48	94.60	37.60	80.90	1.13	39.90	9.70
10676	94.60	0.82	53.80	12.60	75.00	1.44	24.50	7.30
10863	31.40	1.74	10.10	5.10	3.50	1.94	18.20	5.50
11007	85.60	0.58	93.10	42.70	63.90	1.03	58.40	11.90
20070	96.10	0.52	92.80	49.10	81.50	1.21	37.60	9.60
20542	97.40	0.54	95.80	40.30	82.60	1.21	34.80	9.90
20543	97.80	0.52	95.60	47.20	82.20	1.18	35.90	10.60
20544	97.90	0.54	94.50	49.30	78.30	1.22	40.10	10.00
20545	97.80	0.57	90.30	40.50	79.60	1.26	31.90	9.60
20668	79.50	0.72	67.90	18.30	59.10	1.35	29.80	9.20
20672	94.00	0.64	88.90	32.30	72.70	1.27	21.60	8.80
21375	85.60	1.05	48.30	10.50	47.60	1.38	20.80	9.10
21377	80.80	1.15	18.90	7.20	20.30	1.58	7.60	6.70
21391	96.20	0.70	76.70	20.70	65.70	1.32	20.90	7.90
21452	91.60	0.88	70.90	14.40	53.70	1.19	32.60	9.30
21457	91.80	0.88	71.20	15.70	55.60	1.19	31.20	9.50
21864	83.40	0.79	70.90	16.40	1.90	2.19	8.70	11.50
21865	76.60	1.03	43.70	10.20	28.60	1.30	15.60	9.30
21997	83.50	0.77	75.10	19.30	50.00	1.26	32.30	11.30
21999	85.90	0.80	75.30	19.80	41.90	1.23	19.30	10.40
22000	72.00	0.84	53.10	11.30	27.20	1.37	16.50	8.40
22001	85.50	0.92	74.10	16.00	51.30	1.30	35.50	10.00
30037	96.00	0.47	88.60	44.10	84.80	1.04	64.50	11.40
30039	83.20	0.82	79.60	24.60	54.20	1.12	55.50	10.70
30040	86.00	0.63	72.40	26.50	55.70	1.01	53.80	12.00
30127	92.10	0.83	53.60	14.10	53.60	1.21	19.40	9.10
30178	97.00	0.57	87.90	40.60	82.50	1.07	53.90	11.80
30209	87.60	0.74	86.60	36.10	66.00	1.31	33.40	8.70
30210	93.60	0.49	90.70	30.50	77.70	1.07	56.50	10.10
6526	70.90	1.16	39.40	9.00	31.50	1.39	8.00	7.30
6703	82.30	0.86	69.10	14.20	45.40	1.44	12.80	8.40
6704	70.60	1.66	35.20	7.80	28.00	1.65	23.40	8.00
6705	74.50	1.11	46.60	10.40	37.10	1.46	14.20	8.60
6732	79.90	1.07	50.10	9.80	29.30	1.39	9.40	9.30
7063	92.20	0.77	81.00	19.40	66.00	1.44	8.00	7.80
7573	86.60	0.85	79.90	17.70	56.50	1.18	36.30	10.60
7574	69.00	1.36	24.70	7.20	31.60	1.55	6.20	6.50
7575	68.80	1.31	38.10	7.70	24.20	1.41	9.30	8.60
7577	50.60	2.29	52.40	6.90	34.70	2.23	35.80	6.20
7578	62.10	1.47	17.60	6.20	20.90	1.54	4.50	7.00
7631	89.90	0.95	69.80	14.90	37.60	1.48	7.00	6.70
8331	93.80	0.77	85.50	22.50	69.60	1.38	21.50	9.10
8783	68.40	1.28	41.30	8.30	26.30	1.59	10.80	8.00
8784	85.10	0.93	68.70	13.20	53.10	1.40	29.30	8.60
8791	79.20	1.08	39.90	9.30	41.90	1.47	6.20	7.80
9588	85.10	1.23	26.60	7.50	22.80	1.42	9.10	6.80
9589	77.30	1.34	16.40	6.60	31.30	1.59	7.50	7.20
9891	89.80	0.91	64.40	17.20	50.30	1.33	16.10	7.50

Table S3: Results of DeepTracer and Phenix for dataset of 476 cryo-EM maps. Evaluation was accomplished using Phenix's `chain_comparison` tool. For three rows the tool did not return any values for Phenix's prediction and the corresponding cells are filled with a '-'.

	**DeepTracer**				**Phenix**			
EMDB ID	% Matching	RMSD	% Seq Matching	Mean Length	% Matching	RMSD	% Seq Matching	Mean Length
2278	87.60	1.31	64.20	12.70	64.50	1.24	17.10	8.10
2364	59.90	1.80	5.70	4.90	49.40	1.91	9.00	5.40
2513	98.10	0.81	90.30	26.50	62.60	1.17	11.80	6.60
2566	95.20	0.79	84.90	23.00	51.60	0.99	21.00	9.10
2599	77.10	2.05	44.20	7.90	22.80	1.96	9.50	6.30
2650	92.30	0.82	90.40	28.30	59.80	0.98	19.40	9.30
2699	63.30	1.22	60.30	15.80	3.50	1.20	30.30	10.50
2762	92.10	0.88	73.00	17.80	45.80	1.09	15.00	8.10
2763	65.50	1.23	19.30	7.10	26.30	1.33	10.60	7.60
2764	76.00	1.19	28.40	8.30	29.50	1.22	9.00	7.30
2773	74.10	1.43	19.80	7.20	12.60	1.40	8.50	6.60
2807	73.00	1.38	48.90	9.80	44.20	1.34	7.60	7.20
2832	88.70	1.19	78.10	20.10	32.20	1.33	7.80	8.00
2842	93.50	0.89	96.50	20.40	60.80	1.08	23.70	8.50
2847	85.20	0.92	65.70	12.90	20.30	0.84	17.60	9.20
2850	86.80	1.13	36.20	8.40	63.50	1.39	5.90	6.10
2856	77.70	1.10	55.70	10.70	49.70	1.38	7.60	6.80
2857	72.60	1.09	65.40	10.90	45.30	1.22	8.20	6.90
2867	88.30	1.36	67.90	9.80	68.70	1.50	8.50	7.40
2870	52.30	1.58	34.80	7.90	30.60	1.36	4.50	7.60
2871	50.80	1.59	46.70	9.60	38.40	1.70	2.70	7.70
2913	80.90	0.95	46.60	12.10	33.50	1.10	9.10	7.70
2924	78.90	1.10	66.30	14.50	56.70	0.97	10.80	10.30
2974	69.40	1.37	11.00	8.20	52.60	1.48	6.90	7.20
2981	84.30	1.23	13.30	7.00	62.90	1.28	9.80	7.40
2984	96.30	0.63	91.40	36.40	87.90	0.59	56.30	18.00
3013	97.80	0.56	98.20	68.10	68.90	1.02	7.00	7.10
3014	98.20	0.54	97.10	55.30	75.70	0.74	39.20	11.50
3019	63.80	0.87	63.80	14.00	26.10	1.11	8.80	7.60
3047	84.50	0.98	63.20	13.20	38.00	1.10	15.40	8.00
3061	94.90	0.89	92.30	25.20	73.90	1.15	12.10	9.50
3062	33.90	1.75	14.00	6.50	61.20	1.50	6.20	6.80
3063	47.40	1.53	10.90	7.50	66.50	1.52	5.80	6.20
3121	73.90	1.28	37.10	7.30	56.30	1.63	5.20	5.60
3129	94.40	0.61	95.00	56.50	78.90	0.94	25.80	10.80
3130	88.60	0.66	91.00	39.30	71.30	1.20	16.70	8.00
3133	47.50	1.64	11.20	5.90	9.70	1.64	10.30	6.80
3137	82.20	1.62	30.10	6.50	56.70	1.74	7.60	5.40
3151	87.80	0.83	71.00	17.60	34.20	0.98	11.20	8.00
3152	77.30	1.05	24.90	8.20	24.30	1.16	9.60	7.60
3178	83.20	1.30	37.10	9.70	44.90	1.37	9.30	7.10
3218	88.40	0.80	87.50	23.90	49.90	1.10	13.20	7.80
3222	85.30	1.49	42.40	7.30	29.40	1.74	4.20	4.80
3231	91.30	1.05	19.20	10.60	69.10	1.18	6.90	8.00
3236	87.60	1.54	59.50	9.50	11.10	1.67	29.20	12.00
3237	85.00	1.38	12.90	8.60	64.20	1.47	9.90	7.90
3238	70.60	1.40	15.60	7.90	56.10	1.50	7.30	8.00
3239	82.00	1.24	18.90	9.50	63.00	1.34	7.40	8.50
3240	69.80	1.49	13.80	8.10	68.30	1.54	8.50	8.00
3246	90.90	1.09	36.60	12.30	62.60	1.47	9.10	6.70
3295	77.20	0.96	77.10	13.60	64.20	0.97	32.10	9.90
3296	79.50	0.96	78.40	12.00	67.40	0.93	44.40	10.80
3297	88.90	1.11	71.40	12.10	57.80	1.01	16.00	8.40
3298	87.50	1.10	73.50	12.00	60.40	0.94	20.70	8.30
3299	77.20	1.09	75.70	14.20	61.50	1.02	19.00	8.50
3308	85.20	1.12	37.50	8.20	52.90	1.38	5.20	6.60

3337	77.50	1.32	20.20	8.50	49.90	1.39	5.40	6.40
3366	53.20	1.60	10.80	6.40	41.80	1.73	5.50	5.80
3378	50.10	1.37	14.80	6.70	37.00	1.47	7.50	6.50
3385	76.90	1.31	34.90	10.50	51.00	1.26	10.80	9.60
3388	80.90	1.12	63.80	14.30	54.90	1.00	12.50	9.80
3446	82.10	1.16	44.80	11.20	50.20	1.43	6.60	6.80
3447	70.70	1.50	10.20	6.10	31.40	1.64	7.60	5.80
3448	77.90	1.41	16.40	7.10	29.50	1.55	6.80	7.00
3460	71.20	1.23	45.30	10.30	46.80	1.28	12.60	7.60
3489	88.80	0.70	89.70	24.30	51.10	0.79	22.00	10.30
3490	86.20	0.69	87.20	22.10	38.90	0.83	14.20	9.70
3492	83.60	0.88	68.20	14.20	23.00	0.94	15.20	7.80
3493	85.90	0.69	83.20	20.30	36.00	0.85	26.80	9.90
3508	90.40	0.83	78.80	18.10	39.20	0.90	17.40	9.30
3523	62.30	1.70	8.80	6.40	54.50	1.79	7.70	6.20
3524	80.70	1.36	49.20	9.90	60.50	1.48	6.50	6.60
3525	81.70	1.04	35.80	9.80	26.50	1.14	13.70	7.30
3533	69.10	0.95	52.20	10.70	6.10	0.99	12.20	8.10
3538	96.10	1.34	98.60	37.00	85.10	1.56	10.70	7.70
3541	51.00	1.63	9.90	6.40	25.20	1.57	7.00	6.80
3570	87.90	0.91	32.10	8.70	59.80	1.38	6.00	6.40
3571	89.30	1.18	71.30	15.60	39.50	1.48	9.40	8.50
3574	89.60	0.71	88.30	24.00	56.20	0.94	29.40	9.90
3575	84.00	1.11	17.60	6.70	36.90	1.53	6.30	5.00
3583	91.20	1.13	68.40	13.60	67.20	1.18	9.30	10.80
3589	87.20	1.26	25.50	9.00	1.50	1.40	0.00	6.50
3593	70.30	1.01	75.30	16.80	46.40	1.27	14.10	7.50
3601	86.30	1.18	43.80	10.10	50.00	1.29	10.60	7.10
3618	81.80	1.00	55.70	11.30	2.90	1.03	8.50	12.60
3622	28.60	1.83	8.00	6.30	35.30	1.99	4.80	5.50
3624	68.10	1.27	31.20	8.10	5.80	1.24	14.20	7.10
3625	68.50	1.27	30.60	8.50	6.40	1.26	16.00	7.10
3630	96.60	0.71	95.70	35.20	76.50	0.87	32.50	8.40
3631	95.70	0.73	91.40	30.00	71.10	1.13	15.40	8.20
3652	57.90	1.11	67.50	27.90	48.20	1.07	24.90	10.00
3656	75.60	1.30	15.90	6.40	6.10	1.27	9.40	7.80
3695	66.40	1.38	19.90	7.20	31.80	1.47	9.40	6.60
3713	91.60	0.75	89.60	24.10	36.10	0.81	20.60	10.60
3727	30.60	1.63	10.30	6.10	33.00	1.78	8.30	6.10
3730	87.00	0.99	48.20	12.00	23.80	0.97	11.60	7.20
3741	91.80	0.80	62.70	11.20	63.00	0.92	10.90	11.50
3742	98.60	0.72	79.20	36.00	60.30	1.10	11.40	11.00
3743	91.80	0.96	59.70	11.20	4.10	1.53	0.00	3.00
3746	82.60	1.13	28.70	7.10	19.80	1.59	9.80	5.10
3748	85.70	0.86	68.90	13.40	25.10	1.06	13.70	8.00
3750	83.70	0.76	75.10	15.60	29.40	0.87	14.90	8.30
3766	18.80	1.70	9.00	6.80	17.40	1.78	6.40	6.90
3770	57.10	1.32	20.90	7.80	13.50	1.28	6.50	7.20
3785	90.30	1.29	79.70	20.80	42.50	1.74	12.50	6.30
3802	68.20	1.51	9.50	6.10	54.40	1.57	7.30	6.40
3817	71.40	0.88	77.30	16.40	43.50	1.18	11.00	8.40
3824	84.70	1.33	30.10	10.10	63.90	1.23	6.40	10.40
3842	64.80	1.31	19.50	6.60	28.30	1.63	6.00	6.20
3843	61.90	1.28	19.20	6.60	30.80	1.68	9.90	5.90
3847	70.60	1.05	41.40	10.50	34.50	1.18	8.80	7.40
3851	88.10	1.11	37.80	5.30	21.40	1.70	22.20	9.00
3855	79.20	1.39	48.20	10.80	41.00	1.60	6.40	6.30
4015	92.40	1.19	79.70	12.20	54.50	1.26	20.50	10.20
4032	85.40	1.48	18.40	8.30	58.30	1.42	6.30	8.80
4037	56.60	1.49	19.80	8.60	44.40	1.40	9.60	8.50
4038	87.40	1.01	70.10	14.70	50.70	1.15	12.60	8.10
4040	87.30	1.42	27.10	9.40	59.60	1.43	6.30	8.60
4050	82.80	1.20	31.60	8.80	7.40	1.18	10.00	7.50
4052	40.70	1.88	18.40	6.20	35.80	1.90	11.10	5.90
4053	85.00	1.27	10.10	6.90	64.40	1.49	9.30	5.80

4054	89.50	1.28	79.40	7.20	84.20	1.44	84.40	8.50
4055	75.00	1.29	38.10	9.40	31.70	1.37	8.90	7.10
4062	97.80	0.89	75.50	24.50	48.30	1.20	11.50	7.50
4063	99.10	0.65	95.40	48.80	71.40	1.06	13.10	8.00
4071	83.10	1.29	20.10	7.20	27.10	1.37	10.60	7.20
4073	85.80	0.97	54.80	11.10	26.70	1.13	14.30	7.80
4074	37.90	1.43	13.40	6.30	14.30	1.43	7.50	8.10
4076	61.60	1.27	14.90	6.40	17.00	1.35	8.90	8.20
4077	49.00	1.42	15.30	6.30	19.50	1.47	9.20	7.50
4079	70.40	1.29	13.00	6.70	18.50	1.28	9.30	7.80
4080	78.00	1.13	30.50	8.30	24.00	1.23	11.50	8.30
4093	43.20	1.20	44.90	11.60	58.40	1.34	9.50	7.90
4112	79.60	1.22	15.90	6.30	56.50	1.66	7.70	4.50
4114	95.40	0.69	88.40	32.30	72.50	0.95	13.80	8.30
4115	89.90	0.88	42.60	12.40	62.90	1.29	7.10	6.00
4118	48.80	1.54	13.90	6.20	38.00	1.68	6.70	5.80
4121	83.40	1.06	37.20	9.50	8.60	1.08	10.20	6.80
4124	85.50	0.99	56.20	12.00	14.40	1.06	16.30	7.70
4125	84.80	0.98	54.90	11.30	14.80	1.06	12.60	7.10
4128	93.30	1.04	74.70	15.90	67.50	1.18	14.00	9.50
4146	86.80	1.13	69.50	14.20	54.00	1.19	9.90	8.30
4147	82.90	1.07	59.70	13.40	47.40	1.24	8.90	7.60
4148	75.80	1.31	31.90	9.40	38.00	1.35	6.90	6.90
5137	70.50	1.79	10.70	6.40	51.30	1.79	8.80	6.70
5185	94.80	1.05	86.40	13.40	68.40	1.14	13.20	6.60
5415	78.90	1.69	11.80	6.20	50.10	1.79	6.90	5.10
5499	85.90	1.24	21.40	7.40	59.80	1.45	7.70	6.20
5520	18.00	1.87	10.80	6.80	18.70	1.35	6.60	9.60
5600	89.20	1.50	9.20	6.30	68.00	1.59	6.10	6.40
5623	94.10	0.71	95.00	25.10	72.40	0.87	35.60	9.10
5776	45.40	1.59	16.70	9.30	50.30	1.28	6.00	11.50
5777	45.60	1.68	18.10	10.00	54.70	1.32	6.50	10.50
5830	89.00	1.39	39.50	13.50	34.10	1.17	9.70	7.80
5925	94.80	1.06	52.20	23.00	11.30	0.80	27.30	11.00
5995	97.40	0.81	96.40	33.20	72.90	1.12	10.20	8.30
6057	72.20	1.33	30.40	8.10	2.90	1.40	8.10	6.60
6123	65.50	1.99	42.10	19.00	72.40	0.92	90.50	10.50
6124	93.20	0.99	42.20	13.60	44.30	1.28	4.90	6.50
6204	62.90	1.47	23.50	7.60	56.70	1.42	7.70	7.40
6224	94.30	0.65	90.50	36.30	71.90	0.99	19.10	9.20
6239	98.50	0.80	89.50	33.40	74.80	0.99	20.40	8.90
6240	82.10	1.01	87.80	28.50	69.40	0.82	25.50	11.30
6266	64.70	0.97	68.00	17.30	-	-	-	-
6267	76.40	1.68	19.20	8.90	67.00	1.31	5.10	8.30
6270	95.80	0.94	88.10	29.30	10.30	1.04	26.30	19.00
6271	93.70	0.93	81.30	15.60	11.70	1.20	13.30	9.00
6272	97.00	0.71	92.50	24.10	88.90	0.70	73.10	19.60
6310	86.70	1.43	62.20	15.90	39.80	1.50	7.80	12.80
6311	78.50	1.43	28.00	7.50	4.90	1.20	8.60	7.60
6324	84.30	1.06	79.50	15.70	69.40	1.13	9.10	8.20
6337	86.80	1.08	41.30	11.60	59.70	1.37	7.70	7.20
6338	88.00	1.12	48.00	12.90	66.50	1.22	8.10	8.10
6344	79.50	1.12	19.80	10.70	75.10	1.12	6.30	8.70
6345	80.50	1.14	21.00	11.30	79.60	1.32	5.20	9.00
6346	84.80	1.08	55.20	13.80	72.80	1.11	18.10	10.00
6349	97.50	0.76	94.40	37.80	45.90	1.54	6.30	6.90
6350	93.00	0.82	81.90	22.00	41.90	1.70	6.60	5.80
6351	94.50	0.78	89.30	32.50	43.40	1.01	11.00	9.50
6352	98.00	0.85	89.10	26.20	26.70	1.19	8.80	6.70
6353	97.80	0.81	92.90	27.80	12.80	1.24	6.40	6.40
6354	94.70	0.90	70.80	17.30	30.10	1.80	3.10	6.50
6394	96.60	0.94	87.70	24.10	67.90	1.42	12.70	6.80
6398	97.40	0.77	95.00	29.70	51.20	1.33	12.20	6.70
6404	90.70	1.09	72.30	13.50	58.10	1.34	9.80	7.20
6408	97.10	0.83	93.50	28.50	79.30	0.83	24.30	10.40

6413	58.40	1.38	35.50	8.50	21.50	1.34	8.60	7.50
6414	47.50	1.42	53.20	10.50	22.90	1.30	10.70	8.30
6415	38.70	1.75	50.10	9.80	20.10	1.71	13.10	7.60
6416	39.20	1.40	46.90	10.10	20.60	1.43	13.80	8.30
6417	32.40	1.38	41.20	8.70	16.60	1.47	11.70	7.10
6418	48.70	1.49	29.40	8.10	20.80	1.42	8.30	8.10
6425	50.90	1.76	9.40	5.70	43.70	1.84	4.10	4.30
6433	76.60	0.94	19.60	6.30	51.60	1.50	6.30	5.70
6435	89.90	1.00	49.50	9.30	65.70	1.34	9.40	7.00
6455	71.00	1.35	60.10	11.60	53.90	1.11	8.20	11.70
6475	56.00	1.52	11.90	7.80	45.40	1.47	5.60	8.50
6480	46.60	1.35	60.60	13.50	25.80	1.32	8.70	8.10
6481	71.70	1.76	14.50	6.80	26.90	1.65	5.10	8.00
6483	73.20	1.35	22.30	7.50	9.20	1.26	14.80	6.80
6486	79.30	1.20	28.90	8.20	12.20	1.14	16.40	7.30
6487	94.70	0.99	66.90	14.20	60.00	1.33	6.50	6.40
6488	93.30	1.02	74.60	14.00	57.30	1.19	7.90	7.40
6526	71.70	1.22	40.50	9.00	38.00	1.37	9.10	7.60
6534	81.60	1.22	46.00	10.70	49.80	1.33	7.30	7.30
6551	95.40	1.07	86.80	22.20	73.90	1.13	19.80	9.60
6555	100.00	0.56	97.90	95.00	73.20	0.94	17.30	10.70
6559	79.30	1.30	41.90	10.00	14.70	1.29	12.40	7.50
6561	50.40	1.16	50.50	11.90	24.50	1.29	10.50	7.50
6562	50.70	1.20	49.20	11.30	23.80	1.31	9.50	7.60
6563	42.50	1.21	58.70	12.30	23.10	1.17	10.90	8.80
6564	31.50	1.44	39.00	9.10	13.10	1.47	10.80	7.90
6565	29.50	1.24	42.30	11.60	19.20	1.41	10.90	7.90
6566	33.60	1.28	46.80	10.80	14.80	1.38	10.00	7.50
6567	28.80	1.36	40.20	10.00	15.30	1.31	9.00	8.70
6568	20.40	1.34	42.40	9.90	12.20	1.45	8.00	8.40
6569	24.50	1.46	26.50	8.80	11.90	1.56	8.10	6.90
6570	22.30	1.67	25.20	7.60	10.70	1.61	5.70	7.00
6573	37.20	1.22	48.10	11.80	17.80	1.32	11.30	8.30
6580	71.40	1.73	16.50	8.30	60.10	1.67	5.80	7.40
6583	80.60	0.85	80.10	17.50	45.00	0.86	15.60	10.40
6584	73.00	1.32	27.80	7.40	3.20	1.17	13.80	7.80
6615	90.50	0.89	73.50	17.00	38.10	0.96	17.00	8.80
6617	79.80	0.90	79.30	15.70	50.70	0.93	15.60	10.40
6629	59.10	1.13	16.50	7.20	22.90	1.71	6.80	5.00
6630	92.90	0.83	90.20	21.00	66.10	1.05	38.70	9.60
6631	87.30	0.86	83.10	20.60	59.50	1.07	12.90	8.00
6632	82.70	1.14	72.20	12.40	53.40	1.04	14.30	9.10
6633	90.70	0.88	87.10	18.80	67.20	0.97	17.70	9.80
6634	89.10	0.96	72.60	18.40	66.70	0.91	12.70	10.30
6635	88.50	0.84	74.90	19.10	62.70	0.89	36.30	10.70
6637	92.70	0.80	36.60	8.70	72.60	1.41	6.40	7.20
6640	69.90	1.63	10.00	8.40	55.70	1.65	5.20	6.90
6656	92.50	0.98	83.70	14.80	62.70	1.05	15.00	8.20
6667	86.70	0.76	87.20	22.10	43.70	0.82	13.40	9.70
6668	42.80	1.77	8.80	7.10	40.80	1.50	6.70	8.70
6675	73.90	0.66	87.40	38.80	56.10	0.83	20.40	13.90
6676	76.60	0.75	88.20	38.00	54.20	0.93	15.60	10.30
6677	68.20	1.38	51.20	11.60	47.70	1.49	11.50	7.80
6679	66.30	1.44	34.60	8.00	48.90	1.48	7.80	7.80
6685	70.60	1.41	17.00	6.20	21.30	1.79	8.20	5.50
6686	97.90	0.60	95.50	50.60	73.60	1.04	15.80	9.20
6687	96.10	0.88	64.90	16.20	63.70	1.42	9.60	7.20
6688	66.80	1.16	17.70	6.30	41.70	1.46	5.00	5.80
6694	52.70	1.68	11.30	6.70	44.80	1.70	8.10	6.90
6698	85.20	1.64	65.20	11.20	63.10	1.50	8.00	9.80
6699	77.20	1.28	49.30	9.90	36.80	1.10	12.10	8.50
6703	83.60	0.97	68.80	14.00	53.20	1.28	14.60	7.70
6704	69.40	1.70	32.60	7.10	41.10	1.73	8.50	6.70
6705	74.30	1.18	50.50	9.70	14.80	1.26	8.40	7.00
6706	59.20	1.54	27.70	6.60	11.30	1.33	13.20	7.20

6707	59.10	1.59	23.30	7.10	39.50	1.62	7.50	5.80
6709	72.60	1.16	43.20	9.80	8.00	1.08	12.10	7.60
6710	71.50	1.11	33.60	10.80	29.30	1.15	11.00	9.10
6711	87.70	1.02	73.00	14.40	11.70	0.98	14.50	8.90
6721	47.90	1.16	61.60	13.20	25.10	1.19	10.90	7.90
6724	71.20	1.29	33.60	10.70	50.60	1.29	7.40	9.50
6732	79.70	1.12	46.60	9.50	51.20	1.29	12.60	6.90
6741	85.00	0.79	85.00	19.80	53.10	0.72	30.90	10.60
6742	86.40	0.93	76.50	15.30	45.70	0.89	13.70	9.60
6743	90.20	0.96	59.90	11.50	58.30	1.30	12.90	6.60
6744	85.00	1.05	43.70	10.50	55.40	1.22	8.90	8.30
6747	68.40	0.95	73.80	17.70	41.00	1.29	12.80	8.20
6751	98.90	0.66	91.10	44.20	87.10	0.87	43.30	13.00
6752	98.50	0.65	89.50	35.40	80.20	0.93	47.30	11.70
6757	97.20	0.73	90.90	23.20	69.90	1.37	8.80	6.50
6770	87.90	1.25	51.80	12.10	68.50	1.21	8.10	9.10
6771	92.80	1.12	78.80	16.40	56.20	1.17	13.60	7.90
6772	89.90	1.09	72.60	16.80	59.90	1.13	10.20	8.90
6773	68.00	1.19	58.10	11.80	42.40	1.13	9.10	9.40
6774	86.50	0.94	85.60	19.40	61.70	1.02	18.70	9.00
6777	89.90	0.94	86.20	21.80	64.20	0.85	25.00	9.40
6778	94.70	0.82	81.80	19.50	48.40	1.02	21.80	8.80
6780	87.80	0.95	62.30	13.70	38.50	1.13	19.60	8.90
6784	94.00	0.83	81.80	20.20	47.60	0.98	18.20	8.90
6788	89.90	0.99	58.20	12.10	37.20	1.15	15.10	8.20
6789	94.20	0.81	84.10	20.20	46.60	1.02	15.30	9.10
6790	92.40	0.93	58.40	12.90	32.30	1.02	12.10	8.40
7018	88.00	1.10	63.20	13.50	60.90	1.13	10.00	8.80
7019	88.20	1.14	74.50	16.20	62.60	1.04	17.80	8.30
7030	81.30	1.35	27.20	8.20	2.60	1.61	9.30	5.40
7035	67.20	1.53	10.10	5.70	3.20	2.05	5.00	4.00
7036	91.60	1.17	72.90	18.60	63.70	1.13	14.60	11.00
7040	92.70	1.42	26.60	9.70	59.00	1.28	9.90	7.20
7048	76.30	1.07	61.60	12.50	36.90	1.03	27.50	8.90
7049	71.80	1.30	21.80	7.10	26.10	1.37	11.50	6.60
7050	83.80	0.88	69.00	15.90	44.60	1.07	25.20	9.60
7051	81.30	1.03	83.00	23.60	47.30	1.16	28.90	9.30
7052	65.30	1.02	60.30	11.40	30.40	1.29	16.60	6.50
7063	91.00	0.85	77.50	17.20	71.20	1.20	12.60	7.60
7073	75.70	1.21	61.90	13.90	55.30	1.22	9.60	11.20
8000	66.10	0.95	48.80	9.50	5.20	1.14	14.40	9.00
8001	72.10	0.84	77.50	15.50	1.60	0.83	14.30	21.00
8002	64.50	1.65	18.60	6.40	0.30	2.01	16.70	4.00
8003	80.00	0.90	53.20	12.00	12.30	1.02	12.50	9.70
8004	82.40	1.07	55.90	11.60	4.40	0.88	16.10	20.10
8011	84.00	1.46	12.80	6.40	37.80	1.40	7.00	7.30
8012	61.40	1.47	29.90	7.90	28.50	1.22	7.00	9.10
8013	77.70	1.70	7.60	5.60	19.80	1.44	7.70	7.00
8014	91.00	1.18	45.40	12.40	39.60	1.17	8.20	8.30
8015	97.60	0.62	94.30	46.80	79.20	1.10	10.10	12.00
8064	73.10	1.10	56.00	11.30	40.60	1.38	7.50	6.50
8069	92.90	1.14	38.30	10.30	66.70	1.51	5.30	6.10
8072	97.30	0.72	94.90	35.80	84.60	1.07	20.00	9.00
8094	83.50	1.14	18.30	8.10	37.10	1.54	4.40	5.90
8095	92.60	1.00	51.70	12.20	53.60	1.27	7.80	8.70
8099	77.70	0.87	31.70	9.70	52.10	1.45	3.00	6.00
8100	82.20	0.92	32.40	11.10	51.20	1.40	8.60	6.40
8107	80.10	0.92	81.00	19.00	34.20	1.02	13.10	8.70
8116	84.90	1.11	18.80	7.50	25.20	1.37	5.50	6.60
8117	90.80	0.98	82.70	17.00	71.60	0.95	46.00	10.10
8118	90.10	1.06	78.00	17.80	68.80	0.87	13.70	9.30
8119	81.20	1.11	78.40	18.80	58.40	0.85	20.90	11.00
8124	85.30	1.20	40.20	9.80	32.50	1.38	11.40	7.00
8136	55.00	1.03	39.50	10.70	38.10	1.31	8.80	7.30
8137	53.90	1.02	45.00	11.10	36.70	1.26	9.80	7.50

8138	59.80	0.98	44.10	11.60	30.80	1.32	8.90	7.90
8150	90.40	1.16	17.20	10.40	42.60	1.36	6.30	7.90
8162	92.90	0.92	79.10	16.20	68.30	1.22	8.00	6.40
8163	94.50	0.93	72.00	15.70	63.90	1.26	9.80	5.80
8177	37.60	1.31	62.40	11.00	32.90	1.53	5.20	7.60
8178	31.60	1.19	73.80	12.60	28.00	1.34	5.30	7.00
8179	95.30	1.06	73.70	22.50	40.00	1.47	12.10	6.20
8183	83.00	1.27	31.70	7.00	54.20	1.29	11.20	8.40
8184	93.50	0.64	92.00	37.60	73.30	1.00	11.40	9.00
8188	79.40	1.32	30.60	8.20	28.10	1.46	5.90	5.90
8189	95.70	0.58	96.70	34.50	87.40	0.83	63.20	16.10
8191	84.50	0.98	40.70	12.80	71.30	1.04	15.00	7.50
8192	63.20	1.34	15.10	6.80	59.20	1.26	8.90	7.30
8193	73.60	1.21	13.00	7.90	69.50	1.16	10.50	8.60
8194	96.90	0.69	93.00	47.50	78.60	0.79	36.80	9.60
8200	75.20	1.11	48.10	10.10	71.70	1.29	7.80	8.90
8215	53.60	1.34	34.50	8.40	55.40	1.31	4.50	8.50
8240	69.50	1.37	20.90	5.90	35.60	1.51	6.10	5.60
8242	7.90	2.02	10.20	6.40	29.30	1.93	7.10	4.80
8253	91.00	0.86	80.80	16.90	5.90	1.54	4.30	11.50
8266	90.20	0.90	77.20	27.90	22.50	1.34	5.40	8.00
8284	33.00	1.35	11.30	4.90	50.20	1.65	5.00	5.80
8289	82.40	1.57	23.50	7.10	50.70	1.40	7.90	7.00
8298	93.40	0.93	63.70	12.60	64.50	1.72	8.30	5.80
8315	84.40	1.31	59.30	11.20	53.60	1.21	14.10	9.80
8320	96.10	0.91	82.40	19.00	-	-	-	-
8321	94.30	1.06	75.90	15.80	40.40	1.38	6.10	6.10
8322	88.00	1.14	23.70	7.50	21.80	1.22	4.80	7.20
8323	75.20	1.39	10.30	7.30	33.50	1.56	7.40	6.10
8331	94.40	0.87	76.70	20.20	68.60	1.04	12.00	8.60
8334	64.80	1.97	9.10	5.90	47.80	1.93	7.60	6.00
8342	76.30	1.32	51.20	10.10	46.00	1.20	10.60	8.20
8354	96.10	0.75	95.50	40.50	79.70	0.85	24.40	9.20
8361	93.70	0.78	85.80	22.50	58.00	0.83	23.70	10.90
8368	75.20	1.22	19.40	7.50	23.30	1.30	8.70	7.20
8372	78.10	1.42	40.40	9.20	25.20	1.27	6.00	8.80
8373	77.70	1.31	51.40	10.80	44.80	1.31	8.20	8.10
8375	66.50	1.55	29.00	7.80	13.30	1.44	4.90	8.50
8376	53.10	1.54	25.70	8.10	23.30	1.45	5.70	8.20
8377	54.40	1.49	29.70	8.10	13.50	1.45	4.80	8.40
8378	60.00	1.42	39.00	8.90	12.90	1.40	5.20	9.20
8379	63.40	1.44	35.60	8.70	41.00	1.29	5.00	9.30
8380	67.60	1.42	36.50	8.80	6.50	1.28	4.90	9.20
8381	61.90	1.50	30.60	8.10	39.80	1.47	5.70	8.00
8382	71.90	1.39	41.90	9.20	33.40	1.27	5.70	8.90
8387	53.70	1.51	28.50	8.30	42.30	1.43	4.80	7.90
8390	63.40	1.56	19.80	7.80	46.10	1.49	4.60	8.30
8391	66.60	1.42	37.00	8.70	34.70	1.46	7.40	7.40
8395	73.00	1.44	31.60	8.60	45.20	1.41	4.30	8.40
8397	95.80	0.70	84.30	35.00	66.30	1.08	11.90	7.60
8398	94.40	0.87	80.70	18.80	64.80	1.43	5.70	6.40
8399	90.20	0.73	86.50	27.50	68.20	1.12	10.00	8.50
8405	86.60	0.99	29.60	8.90	82.30	1.21	30.40	7.50
8409	91.10	1.36	35.90	12.70	60.70	1.40	10.20	10.00
8410	90.80	1.01	80.20	15.20	67.60	1.07	12.60	8.50
8414	24.80	1.63	12.80	6.30	61.30	1.30	9.20	7.30
8435	91.00	0.99	67.30	14.40	65.70	1.17	11.10	7.60
8454	82.30	1.12	54.40	9.20	65.40	1.31	9.50	7.90
8461	82.20	1.45	55.90	11.10	65.70	1.48	7.10	7.70
8469	71.60	1.73	9.60	8.30	47.70	1.73	9.20	6.80
8477	84.00	1.09	66.50	13.60	31.30	1.24	15.40	7.80
8478	91.50	0.93	83.30	20.70	56.70	1.12	17.50	8.50
8479	47.70	1.64	11.70	7.30	43.30	1.60	6.50	7.20
8481	87.90	1.09	35.20	8.70	44.40	1.30	11.20	7.50
8482	75.90	1.37	20.90	9.30	65.90	1.41	6.00	9.70

8505	53.70	1.81	9.00	5.80	2.50	1.42	8.60	7.20
8506	86.80	1.14	34.10	9.30	7.10	1.16	10.80	7.40
8511	90.90	1.21	73.10	13.30	67.60	1.00	25.20	9.30
8512	95.00	1.10	78.90	17.70	50.60	1.00	21.20	8.20
8515	94.00	0.96	63.40	16.00	76.50	1.19	5.40	8.40
8516	81.10	1.42	46.80	9.50	64.00	1.48	6.00	8.40
8517	78.60	1.34	13.60	8.10	67.70	1.63	6.70	6.80
8540	54.70	1.38	34.50	9.40	38.00	1.50	8.30	7.10
8559	86.00	1.33	61.10	11.50	62.40	1.23	12.00	7.80
8560	88.40	1.43	50.30	9.20	67.80	1.17	14.10	8.90
8574	96.30	0.54	96.20	41.50	72.10	0.90	38.30	10.10
8576	94.80	0.87	80.10	18.30	50.30	0.95	14.20	9.00
8579	7.70	2.05	10.20	6.40	45.10	1.83	7.90	6.00
8598	88.80	0.75	88.30	23.80	72.20	1.09	29.60	8.60
8605	92.90	0.79	89.20	29.50	72.20	1.18	15.00	8.30
8608	84.40	0.92	64.40	12.50	49.30	1.42	6.30	5.30
8623	90.60	1.16	38.80	11.80	67.90	1.34	8.00	7.90
8632	64.10	1.36	56.50	13.10	57.40	1.20	12.20	14.80
8633	47.30	1.43	14.20	15.20	63.30	1.29	21.20	11.10
8637	93.30	1.32	36.10	18.00	80.00	1.37	3.20	15.40
8641	77.90	1.06	47.90	10.00	19.20	1.19	11.40	7.60
8642	90.20	1.34	59.50	10.60	76.30	1.28	10.00	9.40
8643	82.70	1.23	26.50	7.80	48.30	1.40	6.20	6.10
8644	77.80	1.17	14.30	6.40	65.30	1.47	4.90	6.00
8645	63.20	1.27	20.90	7.10	12.70	1.22	10.90	7.30
8650	58.60	1.41	17.40	9.90	62.50	1.26	10.60	9.00
8651	61.30	1.54	13.50	7.80	70.30	1.44	10.20	7.70
8652	52.80	1.56	11.90	8.20	57.40	1.44	6.20	8.20
8653	73.40	1.57	6.60	6.50	58.60	1.70	8.80	6.00
8658	88.10	1.34	41.10	8.70	68.20	1.49	8.60	6.60
8672	56.90	1.88	10.20	6.40	47.10	1.86	7.10	7.20
8697	83.00	1.24	52.60	10.40	59.50	1.37	7.80	7.60
8702	70.80	1.20	54.50	10.20	48.20	1.12	11.40	8.50
8708	88.40	0.86	78.10	16.30	29.10	0.91	16.80	8.10
8712	90.90	1.09	76.40	18.30	64.90	1.22	10.20	7.10
8713	79.80	1.05	47.20	9.80	45.10	1.17	7.00	8.30
8717	88.20	1.04	46.50	9.00	45.20	1.17	7.30	8.10
8732	86.70	1.06	74.60	14.10	49.80	1.20	12.90	8.30
8744	31.00	1.93	11.30	6.50	14.40	1.98	7.60	6.30
8745	22.00	2.07	10.30	5.70	12.60	1.98	8.20	6.70
8746	91.50	1.17	61.30	13.60	13.00	1.08	9.30	10.70
8751	73.60	1.78	9.10	7.10	63.10	1.67	6.20	8.20
8755	92.20	1.12	62.80	14.30	45.40	1.56	8.40	6.00
8756	92.20	1.02	50.90	16.30	52.90	1.45	5.00	6.60
8757	90.70	1.09	56.00	14.80	46.10	1.78	5.20	5.50
8764	92.60	1.05	78.60	14.50	66.60	0.97	42.10	10.80
8767	95.70	0.85	74.40	31.20	42.30	1.45	11.60	11.50
8771	66.90	1.04	59.90	13.10	38.40	1.32	7.80	7.30
8774	48.90	1.33	14.10	7.80	30.50	1.47	6.60	7.30
8775	67.20	1.13	43.50	10.20	37.40	1.40	5.70	7.50
8776	54.50	1.30	18.70	7.80	30.90	1.46	6.40	7.40
8780	70.80	1.68	11.50	7.90	77.00	1.67	9.60	9.40
8782	90.50	1.41	66.50	15.90	66.70	1.38	12.30	8.10
8783	69.20	1.36	34.00	7.70	38.50	1.55	6.80	5.80
8784	84.70	1.06	69.50	12.80	54.10	1.24	6.00	7.90
8790	60.40	1.43	32.90	6.70	32.90	1.63	9.50	5.80
8791	79.10	1.12	46.40	9.40	47.60	1.36	7.50	6.60
8794	81.60	1.49	8.00	7.30	64.30	1.53	6.20	6.60
8795	80.60	1.04	30.40	10.20	66.20	1.50	27.20	7.10
8820	73.20	1.48	18.30	6.90	53.80	1.54	6.70	6.70
8823	79.40	1.32	36.20	9.60	46.90	1.37	6.50	9.40
8827	59.60	1.13	20.40	7.90	28.10	1.39	10.60	7.00
8847	92.70	1.29	63.90	12.20	73.80	1.22	13.50	8.30
8848	87.70	1.45	49.40	12.60	-	-	-	-
8850	89.70	1.23	27.70	11.30	75.20	1.39	11.50	9.50

8852	86.10	1.38	26.90	11.80	69.50	1.47	11.40	11.10
8855	91.40	1.15	30.10	17.60	79.20	1.12	15.50	16.40
8860	77.80	1.17	39.60	13.40	39.40	1.16	11.50	8.70
8881	91.20	1.01	78.00	23.00	68.90	1.03	12.10	8.50
8882	94.30	1.04	78.30	17.80	68.50	1.02	21.90	8.60
8883	92.30	1.01	85.00	19.00	66.70	1.14	28.10	7.60
9400	86.50	1.12	60.60	13.00	57.50	1.20	7.10	8.90
9511	57.10	1.40	12.60	6.50	28.30	1.39	9.50	7.50
9513	79.30	1.08	73.70	18.80	47.80	1.06	12.00	9.00
9514	73.10	1.46	39.20	10.50	51.20	1.48	6.60	7.70
9515	80.00	1.29	43.00	11.20	54.60	1.40	8.50	7.80
9517	89.40	0.83	80.30	21.90	58.70	1.28	11.00	7.30
9518	73.60	1.40	32.00	8.80	48.30	1.46	8.20	7.40
9519	75.60	1.37	34.90	8.40	53.90	1.35	6.00	8.40
9520	66.60	1.49	24.00	7.80	47.90	1.41	6.00	8.90
9524	70.90	1.07	70.30	15.00	40.90	1.10	13.60	8.20
9525	56.70	1.19	66.60	14.00	31.10	1.21	15.40	8.40
9528	54.00	1.46	29.90	8.80	46.40	1.39	5.20	8.40
9529	47.80	1.45	24.10	9.30	40.30	1.33	6.20	9.10
9535	74.50	1.66	14.60	6.10	51.00	1.70	11.70	6.40
9565	96.20	1.05	93.10	27.40	68.60	1.20	10.20	8.60
9566	82.10	1.39	34.40	7.40	25.00	1.30	6.50	9.60
9567	67.80	1.17	23.70	8.10	61.70	1.47	12.00	8.00
9569	87.20	0.88	37.30	10.30	25.50	1.14	15.80	7.00
9570	90.60	1.08	89.00	21.80	67.00	0.97	14.90	10.50
9571	86.70	1.25	35.90	13.60	67.80	1.29	7.80	9.00
9572	86.80	0.99	67.80	12.60	36.40	1.07	8.00	7.70
9575	90.40	1.16	59.70	11.50	36.70	1.52	6.10	6.00
Avg.	76.93	1.18	49.83	14.05	45.73	1.29	12.29	8.16

References

[1] Eric F Pettersen, Thomas D Goddard, Conrad C Huang, Gregory S Couch, Daniel M Greenblatt, Elaine C Meng, and Thomas E Ferrin. Ucsf chimera—a visualization system for exploratory research and analysis. *Journal of computational chemistry*, 25(13):1605–1612, 2004.

[2] Brandon Frenz, Alexandra C Walls, Edward H Egelman, David Veesler, and Frank DiMaio. Rosettaes: a sampling strategy enabling automated interpretation of difficult cryo-em maps. *Nature methods*, 14(8):797–800, 2017.

[3] Genki Terashi and Daisuke Kihara. De novo main-chain modeling for em maps using mainmast. *Nature communications*, 9(1):1–11, 2018.
